# Effect of Sample Preparation on the Detection and Quantification of Selected Nuts Allergenic Proteins by LC-MS/MS

**DOI:** 10.3390/molecules26154698

**Published:** 2021-08-03

**Authors:** Sorel Tchewonpi Sagu, Gerd Huschek, Thomas Homann, Harshadrai M. Rawel

**Affiliations:** 1 Institute of Nutritional Science, University of Potsdam, Arthur-Scheunert-Allee 114-116, 14558 Nuthetal, Germany; sorelsagu@uni-potsdam.de (S.T.S.); thomas.homann@uni-potsdam.de (T.H.); 2 GV-Institut für Getreideverarbeitung GmbH, Arthur-Scheunert-Allee 40/41, 14558 Nuthetal, Germany; gerd.huschek@igv-gmbh.de

**Keywords:** nut allergenic proteins, sample preparation, protein extraction, tryptic digestion, microwave assisted digestion, SDS PAGE, LC-MS/MS

## Abstract

The detection and quantification of nut allergens remains a major challenge. The liquid chroma-tography tandem mass spectrometry (LC-MS/MS) is emerging as one of the most widely used methods, but sample preparation prior to the analysis is still a key issue. The objective of this work was to establish optimized protocols for extraction, tryptic digestion and LC-MS analysis of almond, cashew, hazelnut, peanut, pistachio and walnut samples. Ammonium bicar-bonate/urea extraction (Ambi/urea), SDS buffer extraction (SDS), polyvinylpolypyrroli-done (PVPP) extraction, trichloroacetic acid/acetone extraction (TCA/acetone) and chloro-form/methanol/sodium chloride precipitation (CM/NaCl) as well as the performances of con-ventional tryptic digestion and microwave-assisted breakdown were investigated. Overall, the protein extraction yields ranged from 14.9 ± 0.5 (almond extract from CM/NaCl) to 76.5 ± 1.3% (hazelnut extract from Ambi/urea). Electrophoretic profiling showed that the SDS extraction method clearly presented a high amount of extracted proteins in the range of 0–15 kDa, 15–35 kDa, 35–70 kDa and 70–250 kDa compared to the other methods. The linearity of the LC-MS methods in the range of 0 to 0.4 µg equivalent defatted nut flour was assessed and recovery of internal standards GWGG and DPLNV(d8)LKPR ranged from 80 to 120%. The identified bi-omarkers peptides were used to relatively quantifier selected allergenic protein form the inves-tigated nut samples. Considering the overall results, it can be concluded that SDS buffer allows a better protein extraction from almond, peanut and walnut samples while PVPP buffer is more appropriate for cashew, pistachio and hazelnut samples. It was also found that conventional overnight digestion is indicated for cashew, pistachio and hazelnut samples, while microwave assisted tryptic digestion is recommended for almond, hazelnut and peanut extracts.

## 1. Introduction

Sensitive and selective analytical multi-parameter methods are necessary to evaluate whether food labelling complies with the legal regulations to protect consumers [1,2]. A particular analytical challenge concerns the labelling of allergens, as minimal amounts of allergenic foods are sufficient to cause severe or even fatal allergic reactions. Since the amount of allergen for an allergic reaction depends on specific allergens and the immune system of an individual, there is no legal threshold for allergens in foods except for gluten [3]. For the analytical detection, only recommendations for specific allergenic foods are made ranging between 10 to 100 mg allergenic food/kilogram [4]. 

Among allergenic food, nuts represent a special group causing often severe allergenic reactions and affecting especially children. However, nuts are particularly used in the food industry and are popular both raw and processed because of their nutritional content, taste and texture [5,6,7]. The most commonly used nuts include almond, walnut and peanut. Other important nuts such as pistachio, hazelnut, macadamia, pecan or even cashew are also among the most popular varieties [6,7]. It is estimated that peanut allergy prevalence rates are in the range of 1 and 6%, with up to 20–30% of peanut and tree nut allergies co-existing in the population [8,9]. The majority of nut allergens have been found to be proteins, belonging to the families of 2S albumins, vicilins, legumes and more generally seed storage proteins [7,10]. Previous studies documented solid proof that the 2S albumins are the most relevant nut allergens and highlighted how the structural similarities between the 2S albumins might explain the observed clinical cross-reactivity [7,11]. Table S1 (Supplementary Materials) presents the complete list of the allergenic proteins of almond, cashew, hazelnut, peanut, pistachio and walnut. The data from this Table were compiled from the online database of the World Health Organization/International Union of Immunological Societies “Allergen Nomenclature” (WHO/IUIS; http://www.allergen.org; accessed on 13 June 2021) and the online UniProt knowledge database (UniProtKB, https://www.uniprot.org; accessed on 13 June 2021). During food processing, the proteins including their epitopes i.e. the specific segments of a protein where an antibody binds to a protein can change. There are two types of epitopes as typically reported for nut allergens: continuous epitopes which are linear sequences of amino acids and discontinuous epitopes which are composed of amino acids which are brought into close physical proximity by the folding of the protein [12]. This distinction is important as especially thermal processing of food destroys or may create new structural epitopes, whereas sequence epitopes are usually retained and analytically detectable by various methods [12,13,14,15,16].

Common detection methods for allergens are molecular biological methods such as ELISA (Enzyme-linked Immunosorbent Assay) and real-time PCR. However, as thermal treatment leads to protein denaturation, structural modifications and changes of properties of the targeted analytes and since complex food matrices can interact with the DNA, these methods can lead to an underestimation of allergen traces [12,14,17]. Proteomic tools have become an important alternative analytical option to detect allergens from foods. Especially, liquid chromatography with tandem mass spectrometry has become popular due to its high sensitivity and specificity, thus the possibility to avoid cross-reactivity to other species and the possibility of multiple-allergen analysis even in processed and complex food matrices [18,19]. MS-identified protein biomarkers have successfully been used to quantify allergenic traces in processed food as well as to authenticate ingredients with species-specific markers [2,18,20,21].

The main challenge in establishing LC-MS/MS proteomics analytical methods lies in the sample preparation procedures that allows efficient detection and quantification of analytes in the subsequent mass spectrometric analysis. This is particularly the case when dealing with food/vegetable samples consisting of complex matrices. They usually contain high levels of proteases and secondary metabolites that significantly interfere with the protein extraction, separation and identification [22,23]. Obtaining the best protein quality and yield being the main target of the extraction, selecting an appropriate extraction method according to the type of sample has therefore to be the first step leading to the further analysis [24]. Conventional sample preparation strategies often involve aggressive methods of extraction of recalcitrant proteins and protein solubilization is usually achieved by a combination of physical and chemical methods using detergents [25]. The efficiency of detergents in the extraction and the dissolution of proteins are often refined by additives such as chaotropes (urea and thiourea) [26]. All such chemical compounds are known to interfere during the protein digestion processes (partly inhibiting the enzymatic activity) or during the LC-MS analysis. It is therefore usually essential to remove such compounds prior to the analysis, making the procedure more challenging. The standard and primary protocols generally used for protein extraction from plant tissues are the conventional buffer extraction such as ammonium bicarbonate, Tris-HCl, and sodium phosphate [27,28], TCA precipitation [29,30,31,32], phenol extraction methods [33,34,35], polyvinylpolypyrrolidone (PVPP) extraction [27,28,36], or using the sodium dodecyl sulfate (SDS) buffer [33,37,38]. Doellinger et al. [39] presented a sample preparation method by easy extraction and digestion (SPEED) using neither detergents nor chaotropic agents but pure trifluoroacetic acid for protein extraction. However, these extraction methods using chemicals such as urea, SDS or acids generally denatures proteins and are therefore not used when molecules are to remain in their folded state, especially for the measurement of biochemical activity [32]. The optimal protocol being a method allowing the total extraction of all protein components from the proteome of the samples to be analyzed, keeping contamination by other molecules to a minimum and enabling a high level of reproducibility.

Besides the extraction, the digestion step is also essential in the sample processing for protein analysis by LC-MS. With conventional digestion methods, the enzyme (trypsin)/substrate (protein) ratios are generally kept very low (between 1:30 and 1:50) with reaction times extending over several hours [28,40]. An increase in enzyme concentrations can speed up the digestion process, thus reducing reaction times. However, this may lead to an increase in enzymatic auto-proteolysis, resulting eventually in an alteration of the signal interpretation [41].

The aim of this work was to establish optimized protocols for extraction and tryptic digestion of nut samples, including almond, cashew, hazelnut, peanut, pistachio and walnut, and to develop and validate methods for quantification of their allergenic protein by LC-MS/MS. The potential efficiency of the ammonium bicarbonate/urea extraction (Ambi/urea), SDS buffer extraction (SDS), polyvinylpolypyrrolidone (PVPP) extraction, trichloroacetic acid/acetone extraction (TCA/acetone) and chloroform/methanol/sodium chloride precipitation (CM/NaCl) to extract/precipitate proteins from the selected nut samples was investigated. Conventional and microwave-assisted tryptic were also assessed. The evaluation of the different extraction methods was focused on the comparison of the extracted protein content, the extraction yields and the composition of the extracted proteins by SDS PAGE. Furthermore, an LC-MS/MS method was developed to analyze the allergenic proteins of each of the 6 nut samples. Finally, an individual protein extraction and digestion protocol was established for the analysis of allergenic proteins from almond, cashew, hazelnut, peanut, pistachio and walnut by LC-MS/MS.

## 2. Results and Discussion

### 2.1. Composition of the Extracted Proteins

Initially, our intention was to establish sample preparation for unsalted and unroasted nut samples as reported in the following, before turning to the more complex processed food matrices.

#### 2.1.1. Protein Content According to the Bradford Method

Five extraction methods as indicated in the objectives above were used to extract proteins from nut samples. Prio to the tryptic digestion and the tandem mass spectrometry analysis of the allergenic proteins, the protein contents of the extracts were evaluated according to the Bradford method and the results are consigned in Table 1. The trends were completely different from one sample to another. For example, the almond extracts obtained from the PVPP, SDS and TCA/acetone methods contained similar protein concentrations of about 15 mg/mL while the CM/NaCl extract showed a concentration of only 03.82 ± 0.13 mg/mL. Conversely, peanut extracts obtained by Ambi/urea, CM/NaCl, PVPP and SDS methods showed protein levels between 10.49 ± 0.41 and 12.64 ± 0.38 mg/mL while the use of TCA/acetone precipitation produced an extract with a protein content of only 06.06 ± 0.10 mg/mL. TCA and acetone have been reported to allow the inactivation of proteases as well as phenoloxidases and oxidases, preventing the oxidation of phenol to quinones that would result in the binding of proteins into insoluble complexes [42]. This should theoretically allow a more effective extraction of proteins, which was not the case here with the peanut samples. It was showed that walnuts contained more free and total polyphenols in both the combined raw and roasted samples as well the highest lipoprotein-bound antioxidant activity among the other nuts [43]. The US Department of Agriculture (USDA) reported a total phenols content of 418, 269, 835, 1420 and 1556 mg GAE/100 mg for almond, cashew, hazelnut, pistachio and walnut, respectively [44].

With cashew samples, the protein concentration values of the five extracts were all significantly different (*p* < 0.001); the lowest value being obtained with the CM/NaCl method (08.72 ± 0.18 mg/mL), followed by Ambi/urea extract (10.24 ± 0.10 mg/mL), TCA/acetone precipitation extract (14.96 ± 1.48 mg/mL) and PVPP extract (16.13 ± 0.59 mg/mL). The highest value was produced by the SDS method (19.13 ± 0.69 mg/mL). The SDS extract also yielded the highest protein value with walnut samples, followed by the PVPP method while the other three methods produced extracts with protein levels between 5.02 ± 0.02 and 06.85 ± 0.21 mg/mL. Finally, the PVPP method proved to be more efficient for the extraction of proteins from hazelnut and pistachio samples. However, although with hazelnut samples the difference between PVPP and other extracts was not in all cases significant, with pistachio samples, the differences with other extracts were found to be always significant; ranging from 20 (Ambi/ure extract) to 55% (CM/NaCl extract).

As the use of the five extraction methods resulted in different amounts of extract in terms of volume, it was relevant to evaluate the extracts obtained not only in terms of their protein content, but also in terms of their extraction yield. For this purpose, the crude protein content of each nut sample was initially analyzed by the Kjeldahl method, using as conversion factor N × 6.25. The results expressed as g of protein per 100 g of defatted dry matter are presented in Table 1. Values of 23.7; 20.7; 15.3; 22.3; 29.6 and 18.0 g/100 g defatted dry matter were obtained for almond, cashew, hazelnut, peanut, pistachio and walnut. Based on the starting sample masses as well as the different amounts of extracts obtained (in volume) and their corresponding protein concentration, an estimation of the protein content in mg protein per 100 mg flour was performed, allowing to determine the different extraction yields. The results of these calculations are also shown in Table 1. Overall, the protein extraction yields ranged from 14.9 ± 0.5 (almond extract obtained by CM/NaCl precipitation) to 76.5 ± 1.3% (hazelnut extract obtained by the Ambi/urea method). From these data (see also Figure S1, Supplementary Materials), it clearly appeared that the Ambi/urea extraction method produced higher protein extraction yields for almond (57.3.5 ± 0.1%), hazelnut (76.5 ± 1.3%), peanut (61.5 ± 0.7%) and pistachio (69.2 ± 1.1%) samples. The SDS buffer extraction yielded the best results with cashew (70.2 ± 2.5%) and walnut (65.8 ± 1.6%) samples. Urea has been reported to attach carbamylations artificial modifications to proteins, preventing samples from being lysed and thus beneficial for protein extraction [39].

#### 2.1.2. Sodium Dodecyl Sulphate–Polyacrylamide Gel Electrophoresis (SDS PAGE)

**Figure 1:**
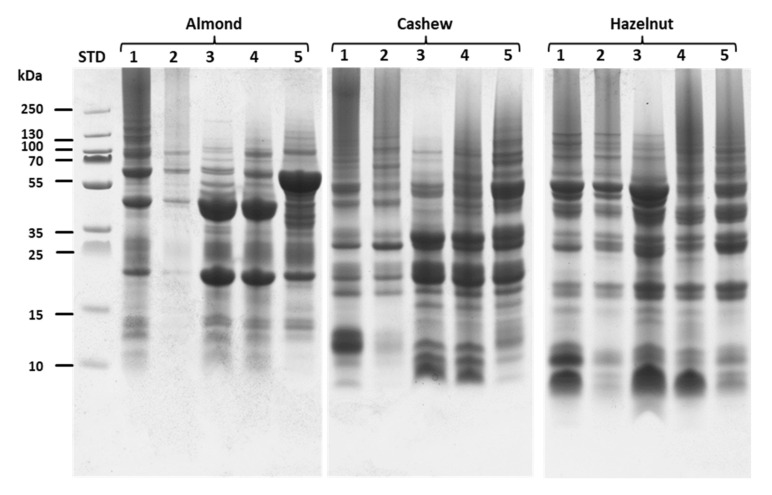
SDS PAGE band profiles of the extracted proteins of almond, cashew and hazelnut. Lines 1, 2, 3, 4 and 5 of the SDS PAGE pictures represent samples from ambi/urea, CM/NaCl, PVPP, SDS and TCA extraction methods, respectively.

**Figure 2:**
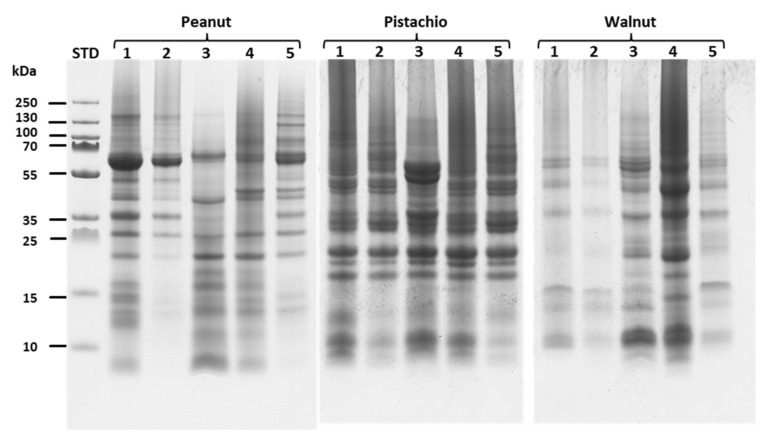
SDS PAGE band profiles of the extracted proteins of peanut, pistachio and walnut. Lines 1, 2, 3, 4 and 5 of the SDS PAGE pictures represent samples from ambi/urea, CM/NaCl, PVPP, SDS and TCA extraction methods, respectively.

The SDS PAGE electrophoresis was performed in order to separate and elucidate the protein composition of the different nut samples according to the applied extraction method and the results are consigned in Figure 1 and Figure 2. To this aim, samples were prepared under denaturing conditions mixing the extracts with the sample buffer in the ratio 1:1 and same volume of the mixture was loaded into the wells of 10% precast polyacrylamide gels for separation. The analysis of the protein composition was performed comparing the protein profiles in different ranges (0–15 kDa, 15–35 kDa, 35–70 kDa and 70–250 kDa) It should be noted that the main allergenic proteins of the different nut samples selected for this study show molecular weights ranging from 5 to 70 kDa (See Table S1, Supplementary Materials).

For almond, if the application of PVPP and SDS extraction methods showed similar profiles with two main protein bands of 20 and 45 kDa, the use of TCA presented a main protein band of 55 kDa. Ambi/urea extract exhibited the presence of these three different bands, but at much lower proportions while CM/NaCl extract presented protein bands of very low intensity (Figure 1). The CM/NaCl method extracted very low amount of protein compared to the other methods while PVPP and SDS extracts showed similar total intensity with similar composition of proteins.

With cashew, PVPP and SDS extract presented similar profiles of proteins as with almond and moreover, it can be seen that their composition in the ranges of 0–15 and 15–35 kDa was almost the same. CM/NaCl, on the other hand, revealed lower protein fractions, especially in the range of 0 to 15 kDa. An approximately analogous pattern was also observed with hazelnut extracts. Here, CM/NaCl precipitation also showed the lowest protein intensity while PVPP and SDS extracts exhibited higher intensities. In view of Figure 1, it is however important to note that the protein profiles of Ambi/urea, SDS and TCA extracts displayed similarities in both areas of 35–70 and 70–250 kDa.

Looking at the peanut extracts, the sample of the ammonium bicarbonate/urea extraction generated the highest number of protein bands, with an intense band at 62 kDa. The extract obtained from TCA/acetone precipitation showed little or almost no protein band with a molecular weight below 25 kDa and it was the same for CM/NaCl extract. PVPP and SDS buffer methods debited similar protein profiles; even though the proteins from the PVPP method seemed to be more abundant (Figure 2). In contrast, it could be shown in the study of Prodic et al [45] that TCA/acetone precipitation of milled peanut grains could results in many proteins in the low molecular weight range depending on the prior treatment executed (e.g. gastric treatments).

Interestingly, when considering the SDS PAGE gel of the pistachio extracts, almost all extraction methods showed a similar profile of extracted proteins, with the exception of the PVPP extract which showed two protein bands of 52 and 56 kDa with significantly higher intensities than the other extracts (Figure 2). However, the PVPP, SDS and TCA/acetone extracts showed a comparable protein configuration in the ranges of 15–35, 35–70 and 70–250 kDa; the PVPP extract, meanwhile, exhibited a significantly higher intensity in the area of the low molecular weight proteins (Figure S2, Supplementary Materials). 

Looking at the walnut samples, it appears that the Ambi/urea and TCA/acetone extracts showed similar profiles while the use of CM/NaCl precipitation resulted in a lower proportion of extracted protein (Figure 2). The SDS extraction method clearly presented a high profile of extracted proteins compared to the other methods, both for low, intermediate and high molecular weight proteins. SDS is an anionic detergent known to bind to proteins through ionic and hydrophobic interactions, solubilising proteins with a wide range of physical properties by changing their secondary and tertiary structures. This leads to optimal extraction of proteins from tissues [46]. However, the presence of SDS in samples raises questions about LC-MS-based proteomics analysis as it can interfere with reversed-phase chromatography separations and electrospray ionization [38], and thus needs to be entirely washed out after extraction. As with peanut, the TCA/acetone extract of walnut samples showed a low protein density in terms of composition compared to SDS, PVPP or Ambi/urea extracts. Similar results were obtained by Yadav et al. [27] who found that after precipitation in 20% TCA-acetone, the amount of soluble protein was lower than in 50 mM Tris-HCl (pH 7.5) and 1.5 M Tris-HCl (pH 8.8) solutions. In contrast, when testing different extraction methods for tomato pollen proteome analysis, Sheoran et al. [47] found the TCA-acetone protein extraction method to be more appropriate. In fact, while TCA/acetone precipitation minimizes protein degradation and protease activity and reduces contaminants such as salts or polyphenols, prolonged incubation of samples in TCA/acetone can lead to protein modification, protein degradation and moreover, precipitated proteins tend to be more difficult to solubilize and require additional NaOH to increase their re-solubilization [30,31,48]. It seems that this extraction protocol may lead to intact sub-units of selected major storage proteins (~50–60 kDa) as shown for almond samples (Figure 1).

### 2.2. LC-MS/MS Evaluation of the Extraction Methods

#### 2.2.1. Method Development and Validation

Nut proteins obtained from the different extraction protocols were further digested and analyzed by LC-MS to assess the efficiency of the extraction methods. For this purpose a method with multiple reactions monitoring (MRM) mode was developed. According to the allergenic protein list of nuts referenced on the online database of the WHO/IUIS Allergen Nomenclature Sub-Committee of the Allergen Nomenclature, 3 allergenic proteins of cashew (11S globulin seed storage protein Ana o 2.0101 Fragment, UniProtKB-Q8GZP6; 2s albumin, UniProtKB-Q8H2B8 and vicilin-like protein, UniProtKB-Q8L5L5) as well 4 individual proteins were selected and analyzed for each nut sample: Cysteine rich antimicrobial protein (A0A516F3L2); prunin 1 Pru du 6.0101 (E3SH28); profilin (Q8GSL5) and (R)-mandelonitrile lyase (Q945K2) for almond, isoflavone reductase (A0A0U1VZC8), 2S albumin (D0PWG29, vicilin Cor a 11.0101 (Q8S4P9) and non-specific lipid-transfer protein Cor a 8.0101 (Q9ATH2) for hazelnut, non-specific lipid-transfer protein (B6CEX8), glycinin fragment (O82580), conglutin-7 (Q6PSU2) and conglutin (Q647G9) for peanut, vicilin Pis v 3.0101 (B4 × 640), Pis v 1 allergen 2S albumin (B7P072), Pis v 2.0101 allergen 11S globulin precusor (B7P073) and Pis v 5.0101 allergen 11S globulin precusor (B7SLJ1) for pistachio, and 11S globulin (A0A1L6K371), vicilin Jug r 6.0101(A0A2I4E5L6), 2S albumin seed storage protein fragment (P93198) and 11S globulin seed storage protein Jug r 4 (Q2TPW5) for walnut. Individual methods were then developed for each nut samples (almond, cashew, hazelnut, peanut, pistachio and walnut).

For each of these proteins, numerous of peptides originating from the tryptic digestion were analyzed and finally, one quantifier (biomarker) and two qualifier peptides were selected for each of the selected proteins. The quantifier peptides were primarily used to compare the relative abundance of the corresponding protein in the samples. The criteria applied for the selection of the biomarker peptides were: the specificity of the peptide, the quality and the stability of the intensity signal of at least 3 peptide fragments (transitions) and where possible, the peptides containing cysteine residues were not selected, this to avoid the structural carbamidomethyl (c) modification performed during the sample preparations. Table S2 (see Supplementary Materials) summarizes the LC-MS/MS optimal conditions for the selected biomarker peptides.

The next step consisted to the validation of the six developed LC-MS/MS methods in order to assess their reliability for the detection and quantification of the selected biomarkers. The linearity measured in the concentration range of 0 to 0.4 µg equivalent defatted nut flour injected, depending on the nut sample and the extraction method, yielded coefficients of determinations *R*^2^ in the range of 0.9703 to 0.9961 (*n* = 3) (see Figures S3–S9, Supplementary Materials). Intra-day and inter-day variations (repeatability and reproducibility) showed relative standard deviations (% RSD) between 0.98 and 8.01% for all the nut samples. The slopes and the intercepts obtained from the linear regression models (data from the linearity) were used to determine the limit of detection (LOD) and the limit of quantification (LOQ) according to the International Conference on Harmonization (ICH). Tables S3–S9 present the complete results obtained for the 23 biomarkers of the nut allergenic proteins selected for this study. The minimum values of the LOD of 35.6, 29.6, 12.3, 9.1, 24.0 and 34.4 ng flour; and of the LOQ of 118.6, 98.7, 41.0, 30.6, 80.1 and 114.8 ng flour were obtained for almond, cashew, hazelnut, peanut, pistachio and walnut, respectively (See Tables S3–S9). All these data demonstrated the efficiency, stability and reproducibility of the different methods developed; fulfilling the validation criteria and thus allowing their use for the relative quantification analysis of the nut allergen proteins selected for this study.

#### 2.2.2. Matrix Effect

**Figure 3:**
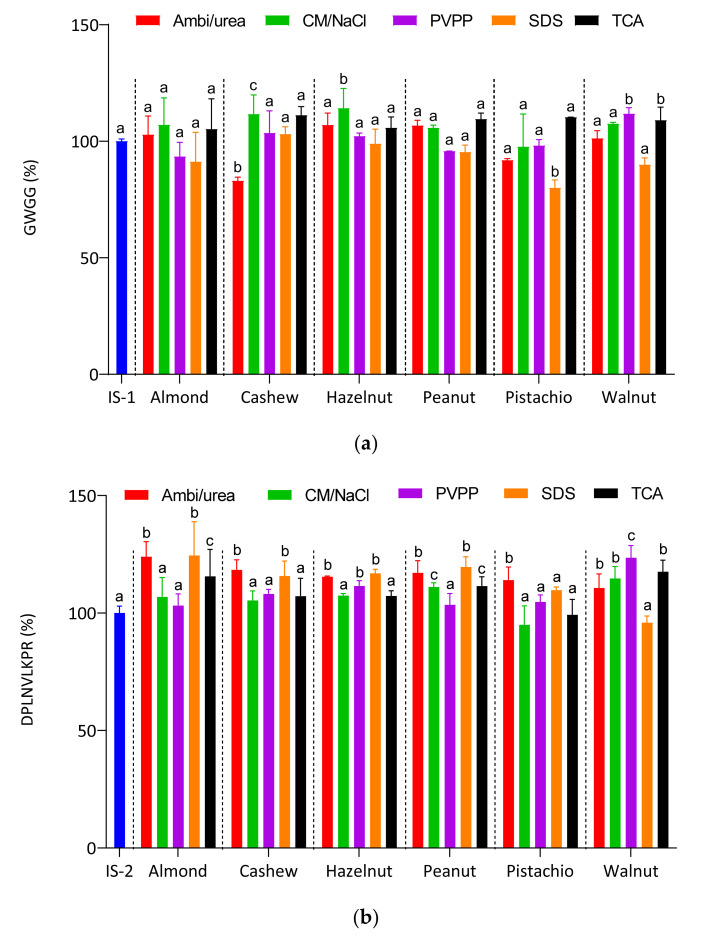
Recovery of the injected internal standards (**a**) GWGG and (**b**) DPLNV(d8)LKPR in the different nut matrixes according to the extraction protocols: ammonium bicarbonate/urea (Ambi/urea), chloroform/methanol/sodium chloride (CM/NaCl), polyvinylpolypyrrolidone (PVPP), sodium dodecyl sulfate (SDS) and trichloroacetic acid (TCA). The results are expressed in percent internal standard detected (*n* = 3). Comparisons were performed per family with internal standards IS1 and IS2 as reference, and different lowercase letters to the standards indicate significant differences at *p* = 0.05 (Tukey test).

The matrix effects of digested nut extracts were examined to assess the impact of different extraction protocols on the detectability of biomarkers. For this purpose, a short-chain peptide containing 4 amino acid residues (GWGG; Q1 376,1615+) and the another one, DPLNV(d8)LKPR (Q1 = 530,3479++, heavy), containing 9 amino acid residues (both obtained from Bachem AG, Bubendorf, Switzerland and from peptide&elephants GmbH, Hennigsdorf, Germany, respectively) were used as internal standard (IS). The recovery rate was determined in the different samples for both IS initial concentrations of 0.01 and 0.1 µg/mL, corresponding to the initial injected masses of 0.1 and 1 ng for IS GWGG and DPLNV(d8)LKPR, respectively. The signal intensity of the peaks of the two IS in distilled water and in the different matrices were evaluated and expressed as ng internal standard detected (Figure S10, Supplementary Materials) and as percentage recoveries (Figure 3). As it can be seen, contrasting results were obtained for each of the matrices. The ideal recovery value being 100%, all data were statistically compared to this reference value for both internal standards. The recovery values of GWGG in the five almond matrices showed no significant difference compared to the IS in water while the recovery values of DPLNVLKPR in the digested samples from Ambi/urea and SDS extracts were significantly higher than the reference (*p* < 0.001).

The cashew extracted by Ambi/urea method presented a significantly lower value than the reference for GWGG (*p* < 0.001) while the values of all other cashew extracts were higher; ranging between 103 and 118%. With hazelnut, if only the CM/NaCl extract presented significantly different results from the reference with GWGG, the recovery values of DPLNVLKPR in digested Ambi/urea (*p* = 0.0017), PVPP (*p* = 0.029) and SDS (*p* = 0.0005) extracts were found significantly different from the reference. Similar results were observed with the peanut and walnut samples. A significant difference in GWGG recovery was only recorded for the pistachio SDS extract (*p* = 0.0001) and the walnut PVPP extract (*p* = 0.0368) while almost all extracts showed significantly different recovery rates from the reference set for the internal standard DPLNVLKPR, except for the peanut PVPP and walnut SDS extracts. As for the pistachio extracts, they were almost all in the average of the reference value for both internal standards GWGG and DPLNVLKPR. 

Overall, it appears that for almost all the matrices, the recovery of the two internal standards ranged from 80 to 120%; the lowest value recorded was 82.9% and the highest 119.6%; with the exception of the recovery of SI DPLNVLKPR in the digested matrices of Ambi/urea (123.9%) and SDS (124.5%) extracts of almond as well as PVPP extract of walnut (123.5%). However, the different results obtained here in term of the matrix effects and the recovery of the internal standards demonstrate the higher sensitivity of the LC-MS system in the detection of analytes, and indicate furthermore the relevance of the sample preparation procedure.

#### 2.2.3. Relative Quantification of Nut Allergenic Proteins by LC-MS/MS

**Figure 4:**
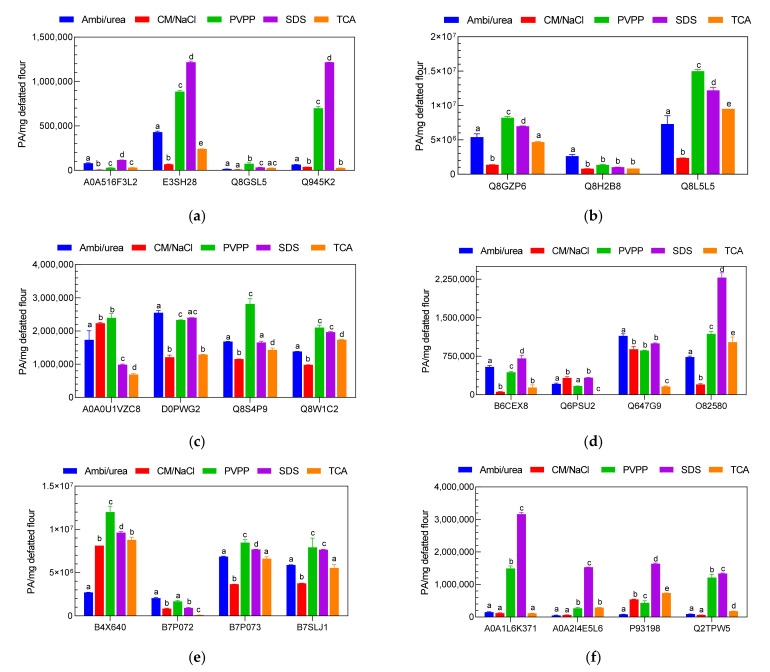
Relative amount of selected allergenic proteins of (**a**) almond, (**b**) cashew, (**c**) hazelnut, (**d**) peanut, (**e**) pistachio and (**f**) walnut expressed as peak area per mg of defatted nut flour according to the different extraction protocols: ammonium bicarbonate/urea (Ambi/urea), chloroform/methanol/sodium chloride (CM/NaCl), polyvinylpolypyrrolidone (PVPP), sodium dodecyl sulfate (SDS) and trichloroacetic acid (TCA). The quantification was performed using the different biomarkers for each protein as determined through method development. Overnight tryptic conventional digestion was performed for all the samples and the experiments were carried out in triplicate (*n* = 3). Comparisons were performed per family and different lowercase letters indicate significant differences at *p* = 0.05 (Tukey test).

The different nut extracts were digested with trypsin and analyzed using the developed and validated LC-MS methods. Quantification of the allergenic proteins of the nuts was then performed using the biomarkers that were identified and selected for each analyzed protein. For this purpose, only a relative quantification of the allergenic proteins was conducted, as the pure 23 biomarkers that could be used as a standard for the calibration required for an absolute quantification were not available. The strategy adopted here consisted in using two different internal standards; a short peptide composed of 4 amino acid residues (GWGG) and a heavy isotope labeled peptide of 9 amino acid residues (DPLNV(d8)LKPR). Accordingly, prior to LC-MS analysis, the two internal standards were mixed with the digested samples to a final concentration of 0.01 and 0.1 µg/mL for GWGG and DPLNV(d8)LKPR, respectively. The ratios between the signal of the internal standards in the samples and those in the double distilled water (IS in sample/IS in Bidest.) were used to normalize the biomarker intensity values. The resulting outcomes are presented in Figure 4.

Three of the four almond proteins analyzed (A0A516F3L2, E3SH28 and Q945K2) showed significantly higher values with the SDS buffer extracts (*p* < 0.001) while the protein Q8GSL5 showed higher values in the PVPP extract. For cashew samples, two of the three proteins analyzed (Q8GZP6 and Q8L5L5) showed higher values in the PVPP extracts and the third protein analyzed, Q8H2B8, was more abundant in the Ambi/urea extract. Comparable results were obtained for hazelnut where the peak values of 2399393 ± 126005; 2816599 ± 162063 and 2105267 ± 74441 PA/mg defatted flour were recorded for the proteins A0A0U1VZC8, Q8S4P9 and Q8W1C2, respectively. With pistachio the values of 1.2 × 10^7^ ± 670153; 8479660 ± 335317 and 7930239 ± 1061258 PA/mg defatted flour were obtained for the allergenic proteins B4X640, B7P073 and B7SLJ1, respectively. SDS buffer extracts gave better results for 3 out of 4 peanut proteins (B6CEX8, Q647G9 and O82580) and all walnut proteins A0A1L6K371, A0A2I4E5L6, P93198 and Q2TPW5. 

For a more comprehensive visualization of these results, the sums of the intensities of the analyzed biomarkers for each nut sample were calculated and the results are shown in Figure S11 (see Supplementary Materials). From this, it appear clearly that the PVPP extraction method produced better results for the cashew, hazelnut and pistachio samples with cumulative peaks of 2.456 × 10^7^ ± 0.403 × 10^6^; 9652624 ± 368183 and 2.925 × 10^7^ ± 2151048 PA/mg defatted flour, respectively, while the SDS buffer extraction method was found to be more efficient to analyze the selected allergenic proteins from almond, peanut and walnut samples. Cumulative values of 2581614 ± 20916; 4322517 ± 166637 and 7679783 ± 86560 PA/mg defatted flour were obtained for almond, peanut and walnut samples, respectively. Overall, these results exhibit a similar trend to those obtained with the extracted protein content and SDS PAGE analysis. The success of the SDS extracts is probably related to its ability to denature the three-dimensional structures of the proteins, allowing the trypsin to reach more efficiently the different cleavage sites on the proteins. The PVPP on the other hand allows the binding of polyphenols during the extraction process, thus releasing the proteins from the complexes structures, making them more accessible.

#### 2.2.4. Effect of the Digestion Method

**Figure 5:**
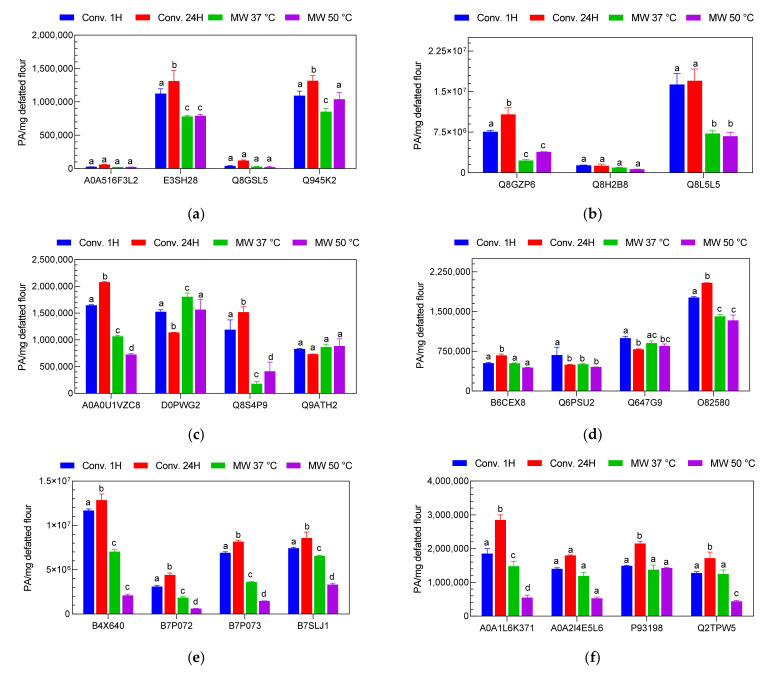
Comparison of the conventional and microwave assisted tryptic digestion on the relative amount of selected allergenic proteins of (**a**) almond, (**b**) cashew, (**c**) hazelnut, (**d**) peanut, (**e**) pistachio and (**f**) walnut expressed as peak area per mg of defatted nut flour. The quantification was performed using the different biomarkers for each protein as determined through method development. Overnight tryptic conventional digestion was performed for all the samples and the experiments were carried out in triplicate (*n* = 3). Comparisons were performed per family and different lowercase letters indicate significant differences at *p* = 0.05 (Tukey test).

Besides the extraction process, protein digestion is one of the key steps involved in the sample preparation process in proteomics. This is especially true since the ultimate analytes used for the LC-MS/MS analysis are the peptides resulting from the degradation of proteins. In this respect, there are several enzymes often used, including trypsin, chymotrypsin, pepsin, pronase and many others. Trypsin, however, is the most commonly used enzyme in proteomics, due to its high specificity and its ability to digest efficiently proteins from different sources and matrixes. In this study, trypsin was used to evaluate two different digestion methods: the conventional digestion method performed at 37 °C in a thermoshaker, and the microwave-assisted digestion method. The conventional method was conducted at two different reaction times: 1 and 18 hour (overnight), while the microwave assisted digestion was conducted for a total reaction time of 11 min at two different temperatures: 37 °C and 50 °C. Only the extracts from the methods that yielded the best results previously were used here (SDS buffer extracts for the almond, peanut and walnut samples, and PVPP extracts for the cashew, hazelnut and pistachio samples. The resulting outputs are presented in Figure 5. 

The first observation is the significantly higher results achieved with both conventional digestion for most of the biomarkers examined, with the exception of hazelnut peptides EVMETAR and CGVNIPYK, and peanut peptides CDLEVESGGR and CDLDVSGGR corresponding to the proteins D0PWG2, Q9ATH2, Q6PSU2 and Q647G9, respectively. For these biomarkers, microwave-assisted tryptic digestion appeared to be more effective. Furthermore, when comparing the conventional digestion carried out during 1 hour and the overnight digestion (18 h), it emerged that significantly improved results were in many cases achieved after 18 h of digestion. Finally, it can be observed that the microwave-assisted digestion was more effective at 37 °C than at 50 °C. 

When considering the sum of the biomarker intensities for each sample, it was found that the conventional digestion performed under the mentioned conditions generated significantly higher intensities with cashew and pistachio samples (*p* < 0.0001). The peak areas obtained were about two times more higher in cashew; conventional digestion for 1 and 18 hours yielding values of 2.528 × 10^7^ ± 148,049 and 2.915 × 10^7^ ± 3,635,161 PA/mg defatted flour, compared to the values of 1.049 × 10^7^ ± 694,536 and 1.128 × 10^7^ ± 734,168 PA/mg defatted flour obtained with microwave-assisted tryptic digestion at 37 and 50 °C, respectively (Figure S12, see Supplementary Materials). With a cumulative biomarker intensities of 8,529,182 ± 400,953 PA/mg defatted flour, conventional digestion at 18 hours generated significantly higher results (*p* > 0.001) with walnut samples, compared to the other digestion types. More surprisingly, looking at Figure S12 (Supplementary Materials), it appears that when the peak areas of all biomarkers for the almond, hazelnut and peanut samples are cumulated, the different tryptic digestion methods (conventional and microwave-assisted) yielded similar results and the analysis of variance revealed that these were not statistically different. 

The results obtained here show that the composition of the extracts may have a significant influence on the digestion process. Therefore, considering all these results, it seems appropriate to say that conventional digestion performed overnight would be the most suitable method to perform the protein digestion of cashew, pistachio and walnut extracts. On the other hand, microwave-assisted tryptic digestion can be recommended for the digestion of almond, hazelnut and peanut extracts. This method being performed in only eleven minutes allows saving time and above all prevents/reduces the probable modifications that may occur during the sample preparation and which occasionally significantly affect the efficiency of detection and quantification of the analytes.

## 3. Materials and Methods

### 3.1. Materials

Unsalted and unroasted nut samples, including almond (*Prunus dulcis* L.), cashew (*Anacardium occidentale* L.), hazelnut (*Corylus avellana* L.), peanut (*Arachis hypogaea* L.), pistachio (*Pistacia vera* L.) and walnut (*Juglans regia* L.) were obtained from a local supermarket (Potsdam, Germany). Soxhlet extraction of fat from almond, cashew, hazelnut and pistachio samples was performed using a SOXTHERM^®^ solid-liquid extraction system, while fat extraction from peanut and walnut samples was performed by the beaker method with petroleum ether as solvent.

Ammonium bicarbonate, tris, tris hydrochloride, sodium dodecyl sulfate (SDS), ethylenediaminetetraacetic acid (EDTA), trichloroacetic acid, sodium chloride, 1,4-Dithiothreitol (DTT), tris(2-carboxyethyl)phosphine (TCEP), glycerol, acetonitrile LC-MS grade, chloroform, methanol, and formic acid were purchased from Carl Roth GmbH (Karlsruhe, Germany). Acetone and n-hexane were obtained from VWR International GmbH, Darmstadt, Germany. Polyvinylpolypyrrolidone was provided by Fluka Chemie GmbH, Buchs, Switzerland and urea was obtained from Merck KGaA, Darmstadt, Germany. Triton™ X-100, iodacetamide (IAA) and 3-(*N*-morpholino) propane sulfonic acid (MOPS) were from Sigma Aldrich GmbH, Steinheim, Germany. The digestion of protein was carried out with a crystalline trypsin, proteomics Grade (Sigma-Aldrich Chemie, Steinhagen, Germany). Short-chain peptide containing 4 amino acid residues - GWGG (Bachem AG, Bubendorf, Switzerland) as well as a stable isotope peptide labeling with 9 amino acid residues DPLNV(d8)LKPR (peptide&elephants GmbH, Hennigsdorf, Germany) were used as internal standards during the LC-MS analysis for the relative quantification purposes. All the other chemicals used in this work were of analytical grade.

### 3.2. Sample Preparation

#### 3.2.1. Extraction Procedures

**Figure 6:**
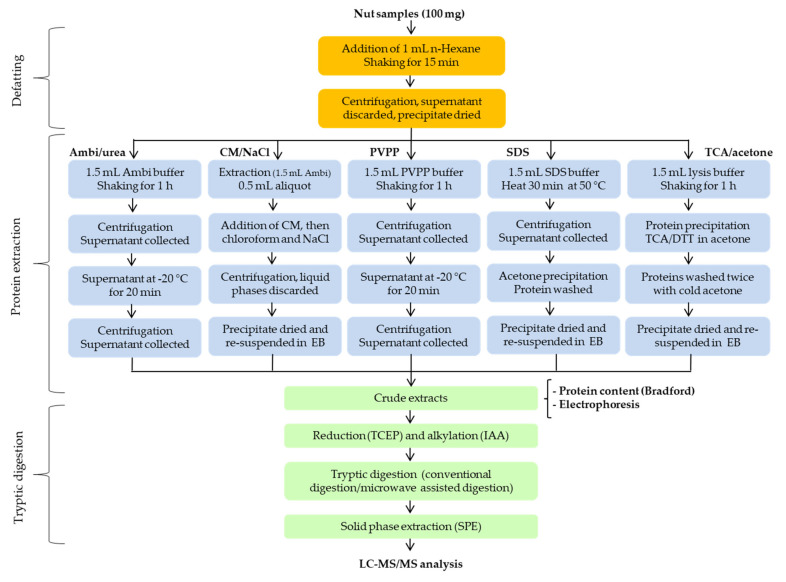
Schematic flowchart of the four protein extraction protocols used. CM is the mixture of chloroform/methanol (ratio 2:1); EB the extraction buffer consisting of 100 mM ammonium bicarbonate and 4 M urea; DTT the reducing agent dithiothreitol; TCEP the reducing agent tris(2-carboxyethyl)phosphine and IAA the alkylation agent iodoacetamide.

Six different extraction methods among those most commonly used, the ammonium bicarbonate/urea extraction, SDS buffer extraction, polyvinylpolypyrrolidone (PVPP) extraction, trichloroacetic acid/acetone extraction and chloroform/methanol/sodium chloride precipitation were applied for the extraction of proteins. Figure 6 presents the schematic flowchart of the extraction protocols. The first step common to all the extraction methods was a second defatting step to remove the residual lipid components. For this purpose, n-Hexane solvent was added to the nut powders (ratio 1:10; *w*/*v*) and the whole was mixed for 20 min at room temperature, then centrifuged at 10,000× *g*, 4 °C for 5 min. The supernatant was removed and discarded, while the precipitate was dried at room temperature in a fume hood for about 1 hour.

##### Ammonium Bicarbonate/Urea Extraction

The extraction protocol using ammonium bicarbonate buffer containing urea is the one optimized and applied in our previous work [28,49,50]. Briefly, 100 mg of each defatted nut sample were mixed with 1.5 mL of extraction buffer (100 mM ammonium bicarbonate containing 4 M urea) and the whole was shaken at 95 rpm at room temperature for 60 min, followed by the centrifugation (10,000× *g*, 4 °C for 5 min). The supernatants were collected into the new eppendorf 1.5 ml eppis, kept at −20 °C for 20 min, after thawing at 4 °C for 20 min, centrifuged (10,000× *g*, 4 °C for 5 min) and the clear supernatants constituting the nut protein extracts were collected and stored at −20 °C for further analysis.

##### Chloroform/Methanol/Sodium Chloride Extraction

Chloroform/Methanol/sodium chloride (CM/NaCl) extraction is a three phase partitioning concentration and purification method, allowing the precipitation of proteins from aqueous solution. Here, 100 mg of defatted nut samples were first extracted with extraction buffer according to the ammonium bicarbonate/urea method as described previously (using 1.5 mL of 100 mM ammonium bicarbonate containing 4 M urea). Volumes of 0.5 mL of the obtained clear supernatants were collected into the 2 ml eppis, 0.8 mL of chloroform/methanol mixture (1:2, *v*/*v*) was added in each, and the whole was vortexed for ca. 1 min. Another 0.5 mL of 100% chloroform solvent was added and the mixture vortexed again for 1 min. Finally, 0.2 mL of NaCl solution (1:10, *w*/*v*) was introduced and the mixture vortexed again. The samples were then incubated for 10 min at 4 °C, which resulted in the formation of the 3 phases: the upper phase consisting of the organic solvent, the lower aqueous phase and the intermediate phase consisting of the protein precipitate. For enhancing the phase separation, the samples were further centrifuged at 2000× *g* for 10 min, and then the upper and lower phases were carefully removed and discarded. The precipitates were dried at room temperature for 2 hours in a fume hood, then re-dissolved in 0.5 mL of the extraction buffer (100 mM ammonium bicarbonate containing 4 M urea), incubated overnight at 4 °C. Finally the samples were vortexed and sonicated for 5 min for complete protein re-dissolution followed by the centrifugation (10,000× *g*, 4 °C for 5 min). The supernatants from the protein extracts were collected and stored at −20 °C.

##### Polyvinylpolypyrrolidone Extraction

The extraction method using polyvinylpolypyrrolidone (PVPP) consisted of adding 1.5 mL of PVPP extraction buffer (0.2 M 3-(*N*-morpholino) propane sulfonic acid (MOPS) pH 7.0, 5% PVPP, 1% triton X-100, 10% glycerol, and 2 mM DTT) to 100 mg of each sample, followed by mixing at 95 rpm for 60 min at room temperature. The mixtures were then centrifuged at 10,000× *g*, 4 °C for 5 min. The supernatants were collected, placed at −20 °C for 20 min and then thaw at 4 °C for 20 min, shortly vortexed before being centrifuged at 10000× *g* (4 °C) for 5 min. The clear supernatants constituting the protein extracts were collected in new microtubes and stored at −20 °C for further analysis.

##### Sodium Dodecyl Sulfate Buffer Extraction

Sodium dodecyl sulfate (SDS) is an anionic detergent that allows a high solubility of challenging proteins through both ionic and hydrophobic binding, modifying the three-dimensional structures of proteins. For this purpose, proteins were extracted from 100 mg of each defatted sample by adding 1.5 mL of SDS buffer (4.0 g SDS, 0.61 g Tris, 130 mM Dithiothreitol in 100 mL H2O; the pH adjusted to 6.8 with 0.1 N HCL). The mixture was heated at 50 °C for 20 min on a thermo-shaker (Thermomixer Comfort, Eppendorf-Netheler-Hinz GmbH, Hamburg) with a stirring function (1000 rpm), and then centrifuged at 10,000× *g* for 5 min. The supernatants were transferred to fresh microtubes and 1 mL of cold acetone was added, vortexed and incubated at −20 °C for 20 min. After centrifugation (at 7000× *g*, 4 °C, 5 min), the supernatants were removed, the precipitate rinsed with another 1 ml cold acetone. The whole was further centrifuged under the same conditions and the supernatant discarded. The precipitates were dried under a fume hood for about 30 min. The extracted proteins (precipitate) were taken up in 0.8 ml of extraction buffer (100 mM ammonium bicarbonate containing 4 M urea). The whole was vortexed for 1 min followed by sonication for 5 min to completely dissolve the proteins. After centrifugation at 10,000× *g* for 5 min at 4 °C, the supernatants were collected and kept at −20 °C for the further experiments.

##### Trichloroacetic Acid/Acetone Precipitation

TCA/acetone extraction experiments were carried out as described by Mechin et al. [42] with some modifications. One hundred milliliters defatted nut samples were homogenized in 1.5 mL of extraction buffer (50 mM Tris-HCl, 25 mM sucrose, 0.1 M EDTA pH 8.1% Triton X 100, 10% Glycerol and 10mM DTT). The extraction was performed at room temperature for 1 h and after centrifugation (10,000× *g*, 4 °C for 5 min), the supernatants were collected into the fresh microtubes. For the TCA/acetone precipitation, 1 mL of 10% TCA in acetone containing 10 mM DTT was added to the obtained extracts (for a final concentration of TCA of ~5% in the mixture), the whole was vortexed and then incubated at –20 °C for 1 h. samples were then centrifuged (10,000× *g*, 4 °C for 10 min) and the supernatants were discarded. The precipitates were then washed by adding 1 mL of ice cold acetone, incubated at −20 °C for 20 min, then centrifuged at 10,000× *g*, 4 °C for 10 min. The supernatants were discarded; the precipitates dried for 30 min at room temperature under the fume hood and re-dissolved in 0.7 mL of extraction buffer (100 mM ammonium bicarbonate containing 4 M urea). After vortex and sonication for 10 min, the samples were centrifuged and the clear supernatants consisting of extracted proteins were collected and save at −20 °C for the further experiments.

#### 3.2.2. Tryptic Digestion

Both conventional and microwave digestion were applied and compared. Prior to the tryptic digestion, 400 µL of each extracted nut proteins were first reduced by applying 10 µL of 0.25 M of tris(2-carboxyethyl)phosphin (TCEP) for 20 min at 50 °C, followed by the alkylation (using 10 µL of 0.25 M of iodoacetamide solution for 20 min at 50 °C in the dark), and finally 135 µL of digestion buffer (ammonium bicarbonate) were added. Then, 20 µL of 4 mg/mL trypsin solution were added. The conventional digestion consisted to keep sample for 1 h and overnight (18 h) at 37 °C under the shaking conditions on a thermomixer. The microwave digestion was performed using a CEM Discover SP-D Digestion Microwave (CEM GmbH, Kamp-Lintfort, Germany). Here, the digestion was performed at a pressure of 40 psi with a maximum power of the microwave set at 300 Watt. Two different temperatures (27 and 50 °C) were evaluated with a ramp and hold times of 1 and 10 min, respectively (total digestion time of 11 min). Both conventional and microwave digestions were stopped by adding 15 µL of 40% formic acid.

#### 3.2.3. Solid Phase Extraction

To purify the peptides from the matrix compounds, a solid phase extraction using columns containing 300 mg of C18 material (Chromabond C18 ec, Macherey Nagel Düren, Germany) was performed. The columns were first activated using 6 mL of buffer containing 50% acetonitrile, 50% double-distilled water (bidest water) and 0.1% formic acid; and then conditioned with 6 mL of bidest water. The samples from the tryptic digestion were then loaded onto the columns, washed with 6 mL of bidest water and the peptides were collected using 6 mL of 100% acetonitrile containing 0.1% formic acid. The eluate were finally diluted in a ratio 1:5 (*v/v*) using bidest water and the samples were filled into the vials for the LC-MS/MS analysis.

### 3.3. Analysis

#### 3.3.1. Protein Content

The Bradford method [51] was used to determine the protein concentration of the different extracts. Briefly, 40 µL of adequately diluted protein samples were mixed with 1.6 mL of Coomassie dye binding protein assay (Bradford reagent, Sigma-Aldrich, Steinheim, Germany) and incubated at room temperature for 5 min. The absorbance was then measured at a wavelength of 595 nm using a UV-VIS spectrophotometer (Hanna instruments, Vöhringen, Germany). Bovine serum albumin (BSA) solutions with concentrations of 0 to 1.4 mg/mL were prepared and used as standard for the calibration curve.

The results were expressed as mg protein per mL extract. Furthermore, in order to evaluate the efficiency of the different extraction methods, the extraction yields were determined and reported as µg extracted protein per mg of starting nut powder.

#### 3.3.2. Sodium Dodecyl Sulfate Polyacrylamide Gel Electrophoresis (SDS PAGE)

The sample preparation for SDS PAGE was performed as previously described [52]. Briefly, samples from different extraction methods were mixed in a ratio of 1:1 (*v/v*) with the sample buffer (consisting of 125 mM Tris buffer, pH = 6.8, 20% (*v*/*v*) glycerol, 2% (*v*/*v*) 2-mercaptoethanol, 2% (*w*/*v*) SDS, 0.1% (*w*/*v*) bromphenol blue) and the mixtures were heated at 95 °C for 5 min. After cooling to room temperature, the same volume of the mixture (10 µL of each sample)was loaded into gels (Invitrogen NuPAGE 10% Bis-Tris protein gel, 12 wells, Thermo Fisher Scientific, Carlsbad, CA, USA). Spectra™ Multicolor Broad Range Protein Ladder with molecular weight proteins of 10, 15, 25, 35, 40, 50, 70, 100, 140 and 260 kDa (Thermo Fisher Scientific) was used for calibration purposes. The gels were then run initially at 20 mA for 30 min, and then the current was increased to 50 mA till the end of the separation. The gels were stained overnight with a coomassie blue solution (constituted of one PhastGel^®^ blue R250 tablet in 2 liters of 10% acetic acid), then distained with a 10% acetic acid solution. The distaining solution was changed regularly till for about 3 h. The gels were finally scanned (Bio-5000 Professional VIS Gel Scanner, SERVA Electrophoresis GmbH, Heidelberg, Germany) and analyzed using Image Lab software (Bio-Rad Laboratories Ltd., Hemel Hempstead, UK).

#### 3.3.3. LC-MS/MS—Method Development and Analysis

The allergenic proteins of almond, cashew, and hazelnut, peanut, pistachio and walnut were identified from the online database “allergen nomenclature” of the World Health Organization and International Union of Immunological Societies (WHO/IUIS; http://www.allergen.org; accessed on 13 June 2021). Table S1 (Supplementary Materials) presents the list of all nut allergenic proteins identified and their characteristics. The protein sequences (primary structure) of the different allergens were extracted from the UniProt online knowledge base (UniProtKB) (https://www.uniprot.org; accessed on 13 June 2021) as FASTA files and imported into the Skyline software MacCoss Lab Software, version 20.2, University of Washington, Seattle, WA, USA; https://skyline.gs.washington.edu; accessed on 13 June 2021) [53]. Protein sequences were then theoretically digested under the following conditions: Digestion enzyme-trypsin; Partial-0; Chemical modification-carbamidomethyl of cysteine residues, precursor charges-2 and 3; ionic charge-1; peptide length filter-4 and 30 amino acid residues. All the generated peptides were screened for their specificity using the “unique peptide” function. The peptides were then analyzed and finally 2 to 3 peptides were selected to characterize each allergenic protein.

The allergenic proteins identified in the previous step were tested for their species-selectivity with the BLAST algorithm (Basic Local Alignment Search Tool from the database UniProt Universal Protein Resource, http://www.uniprot.org/; accessed on 13 June 2021). Candidates with less than 95% protein sequence similarity to other species were in-silico digested with the Skyline software using trypsin as enzyme. The additional settings are described in our previous works [28,49,50]. The species selectivity of the identified individual nut peptides was analyzed once more by the BLAST algorithm (UniProt Universal Protein Resource, http://www.uniprot.org/; accessed on 13 June 2021).

The trypsin-digested nut proteins were analyzed with a High-performance liquid chromatography (a 1260 Infinity Binary Pump, a 1260 Infinity II Multisampler, a 1260 Infinity II Multicolumn Thermostat, a 1260 Infinity II Diode Array Detector and an Agilent instrument control framework) coupled with an electrospray ionization tandem mass spectrometry system equipped with an Agilent 6470 Triple Quadrupole LC/MS (1260 Infinity II LC System; Agilent Technologies, Waldbronn, Germany). The HPLC was fitted with a Kinetex^®^ reverse phase C8 column, 2.6 µm, 100 Å, 150 × 4.6 mm (Phenomenex Ltd., Aschaffenburg, Germany). The separation was performed with a column temperature of 30 °C for 28 min with a column equilibration time between each run (post run) of 4 min (total time of 32 min per run); and in gradient mode using 0.1% formic acid in water as eluent A and acetonitrile as eluent B. The elution program was designed as indicated below: 0.01–2.0 min, 5% solvent B; 2–18 min, 50% sol-vent B; 18–19 min, 95% solvent B; 19–22 min, 95% solvent B; 22–23 min, 5% solvent B; and 23–28 min, 5% solvent B. The temperature of the desolvation gas in the ionization source was set at 250 °C with a gas flow rate of 11 L/min and a nebulizer pressure of 35 psi; using nitrogen as the collision gas. The fragmentor voltage and collision energy were individually optimized for every peptide of each nut sample, using a dwell time of 20 ms. Finally, a multiple reaction monitoring (MRM) method to optimally detect specific transitions of the analyzed peptides with previously identified retention times was established. Three to four transitions with the highest intensity response in the product ion spectrum of the respective marker peptides were selected and the relative quantification was achieved by summing the total peak areas of the individual transitions using the software Skyline.

### 3.4. Statistical Analysis

The LC-MS/MS method was validated while applying the linearity, the selectivity, the sensitivity, the recovery, the repeatability and the reproducibility. In addition, the limits of detection (LOD) and the limit of quantification (LOQ) were calculated. All validation tools were performed according to the International Conference on Harmonization (ICH). All the experiments were performed in triplicate and data were expressed as means with their standard deviations. A two-way analysis of variance (ANOVA) was applied followed by Tukey’s test using GraphPad Prism 6 (GraphPad Software, San Diego, CA, USA). The differences between the values were considered significantly different for *p*-values < 0.05 (95% confidence level).

## 4. Conclusions

In this work, five protein extraction methods including the ammonium bicar-bonate/urea extraction (Ambi/urea), SDS buffer extraction (SDS), polyvinylpolypyrrolidone (PVPP) extraction, trichloroacetic acid/acetone extraction (TCA/acetone) and chloroform/methanol/sodium chloride precipitation (CM/NaCl) were tested to extract proteins from almond, cashew, hazelnut, peanut, pistachio and walnut samples. In addition, the performances of conventional tryptic digestion and microwave-assisted tryptic digestion of protein extracts were compared. The objective was to establish optimized protocols for extraction and tryptic digestion of nut proteins, and to develop and validate methods for quantification of allergenic protein contents of each nut sample by liquid chromatography tandem mass spectrometry (LC-MS/MS). The results of the extracted protein content as well as the protein yields and electrophoresis showed that the SDS buffer extraction method was the most suitable to perform the protein extraction from almond, peanut and walnut samples while the PVPP extraction method produced better results for cashew, pistachio and hazelnut samples. These results were confirmed by the relative quantification of allergenic proteins from the digestion of the different extracts by the LC/MS method. It was also found that the conventional overnight digestion was the most effective method to digest cashew, pistachio and hazelnut proteins, while microwave-assisted tryptic digestion was recommended for the digestion of almond, hazelnut and peanut extracts. Further experiments will consider the absolute quantification aspects to allow comparison with other similar studies and to consider selected food processing matrix effects (e.g. backing/roasting, boiling, high pressure treatment etc).

## Figures and Tables

**Table 1:** Composition of nut samples in term of crude protein, defatted dry matter (DDM) total fat, extracted protein contents according to the Bradford method and yields according to the different extraction methods.

		Almond	Cashew	Hazelnut	Peanut	Pistachio	Walnut
* Crude Protein (g/100g)	-	23.7	20.7	15.3	22.3	29.6	18.0
**Defatted Dry Matter (%)**	-	48.2	48.2	33.8	47.9	49.1	41.3
**Total Fat (g/100g)**	-	54.7	46.9	65.3	51.5	51.2	65.6
** Extracted Proteins **(mg/mL)**	Ambi/urea	12.93 ± 0.01 ^a^	10.24 ± 0.10 ^a^	11.94 ± 0.20 ^a^	12.24 ± 0.14 ^a^	19.51 ± 0.31 ^a^	06.77 ± 0.48 ^a^
	CM/NaCl	03.82 ± 0.13 ^b^	08.72 ± 0.18 ^b^	10.81 ± 0.10 ^b^	10.49 ± 0.41 ^b^	11.32 ± 0.05 ^b^	5.02 ± 0.02 ^b^
	PVPP	15.40 ± 0.31 ^c^	16.13 ± 0.59 ^c^	12.06 ± 0.17 ^a^	11.86 ± 0.52 ^a^	24.38 ± 0.03 ^c^	10.20 ± 0.03 ^c^
	SDS buffer	14.89 ± 0.09 ^c^	19.13 ± 0.69 ^d^	11.51 ± 0.14 ^ab^	12.64 ± 0.38 ^a^	15.25 ± 0.17 ^d^	15.18 ± 0.38 ^d^
	TCA/Acetone	15.06 ± 0.06 ^c^	14.96 ± 1.48^e^	12.99 ±0.10 ^c^	06.06 ± 0.10 ^c^	15.97 ± 0.10 ^d^	06.85 ± 0.21 ^a^
**Extraction Yield** **(%)**	Ambi/urea	57.3.5 ± 0.1 ^a^	63.3 ± 0.6 ^a^	76.5 ± 1.3 ^a^	61.5 ± 0.7 ^a^	69.2 ± 1.1 ^a^	37.6 ± 2.7 ^a^
	CM/NaCl	14.9 ± 0.5 ^b^	38.9 ± 0.8 ^b^	65.3 ± 0.6 ^b^	43.5 ± 1.7 ^b^	35.4 ± 0.1 ^b^	25.8 ± 0.1 ^b^
	PVPP	48.7 ± 1.0 ^c^	62.3 ± 2.3 ^a^	48.1 ± 0.7 ^c^	45.2 ± 2.0 ^b^	53.5 ± 0.1 ^c^	48.2 ± 0.2 ^c^
	SDS buffer	46.5 ± 0.3 ^c d^	70.2 ± 2.5 ^c^	57.2 ± 0.7 ^d^	42.5 ± 1.3 ^b^	40.2 ± 0.5 ^d^	65.8 ± 1.6 ^d^
	TCA/Acetone	43.2 ± 0.2 ^d^	49.1 ± 4.9 ^d^	57.7 ± 0.5 ^d^	18.5 ± 0.3 ^c^	36.7 ± 0.2 ^b^	25.9 ± 0.8 ^b^

* Total protein content according to Kjeldahl. ** Extracted protein according to Bradford and expressed as mg protein/mg defatted nut flours. Means with different lowercase letters in the same column indicate significant differences at *p* = 0.05 (Tukey test); *n* = 3.

## Data Availability

Not applicable.

## Supplementary Materials

Figure S1: Extracted protein (a) and protein extraction yield of nut samples almond, cashew, hazelnut, peanut, pistachio and walnut using Ambi/urea, CM/NaCl, PVPP, SDS and TCA methods; Figure S2: Relative composition of extracted proteins of (a) almond, cashew, hazelnut, and (b) peanut, pistachio and walnut using Ambi/urea, CM/NaCl, PVPP, SDS and TCA methods; Figure S3: Linearity of the HPLC-MS/MS measurements of internal standards (a) GWGG and (b) DPLNVLKPR; Figure S4: Linearity of the HPLC-MS/MS measurements of analyzed almond allergenic proteins (a) Cysteine rich antimicrobial protein (A0A516F3L2), (b) Prunin 1 Pru du 6.0101 (E3SH28), (c) Profilin (Q8GSL5) and (d) (R)-mandelonitrile lyase 2 (Q945K2); Figure S5: Linearity of the HPLC-MS/MS measurements of analyzed cashew allergenic proteins (a) 11S globulin seed storage protein Ana o 2.0101 Fragment (Q8GZP6), (b) 2s albumin (Q8H2B8) and (c) Vicilin-like protein (Q8L5L5); Figure S6: Linearity of the HPLC-MS/MS measurements of analyzed hazelnut allergenic proteins (a) Isoflavone reductase (A0A0U1VZC8), (b) 2S albumin (D0PWG2), (c) Vicilin Cor a 11.0101 (Q8S4P9) and (d) Non-specific lipid-transfer protein Cor a 8.0101 (Q9ATH2); Figure S7: Linearity of the HPLC-MS/MS measurements of analyzed peanut allergenic proteins (a) Non-specific lipid-transfer protein (B6CEX8), (b) Conglutin-7 (Q6PSU2), (c) Conglutin (Q647G9) and (d) Glycinin Fragment (O82580); Figure S8: Linearity of the HPLC-MS/MS measurements of analyzed pistachio allergenic proteins (a) Superoxide dismutase (B2BDZ8), (b) Pis v 1 allergen 2S albumin (B7P072), (c) Pis v 2.0101 allergen11S globulin precusor (B7P073) and (d) Pis v 5.0101 allergen 11S globulin precusor (B7SLJ1); Figure S9: Linearity of the HPLC-MS/MS measurements of analyzed walnut allergenic proteins (a) 11S globulin (A0A1L6K371), (b) Vicilin Jug r 6.0101 (A0A2I4E5L6), (c) 2S albumin seed storage protein Fragment (P93198) and (d) 11S globulin seed storage protein Jug r 4 (Q2TPW5); Figure S10: Recovery of the injected internal standards (a) GWGG and (b) DPLNV(d8)LKPR in the different nut matrixes according to the extraction protocols Ambi/urea, CM/NaCl, PVPP, SDS and TCA; Figure S11: Sum of the relative amount of selected allergenic proteins of (a) almond, (b) cashew, (c) hazelnut, (d) peanut, (e) pistachio and (f) walnut expressed as peak area per mg of defatted nut flour according to the different extractions protocols; Figure S12: Sum of the relative amount of selected allergenic proteins of (a) almond, (b) cashew, (c) hazelnut, (d) peanut, (e) pistachio and (f) walnut expressed as peak area per mg of defatted nut flour according to the different digestion protocols; Table S1: List of nut allergenic proteins available from the online database allergen nomenclature, the WHO/IUIS Allergen Nomenclature Sub-Committee (http://www.allergen.org; accessed on 13 June 2021); Table S2: The optimized conditions of the HPLC-MS/MS method for the analysis and relative quantification of almond, cashew, hazelnut, peanut, pistachio and walnut allergenic proteins; Table S3: LC-MS/MS method validation results of almond allergenic proteins; Table S4: LC-MS/MS method validation results of cashew allergenic proteins; Table S5: LC-MS/MS method validation results of hazelnut allergenic proteins; Table S6: LC-MS/MS method validation results of peanut allergenic proteins; Table S7: LC-MS/MS method validation results of pistachio allergenic proteins; Table S8: LC-MS/MS method validation results of walnut allergenic proteins; Table S9: LC-MS/MS method validation results of two peptides used as internal standards.
