# Supplementary material for: Effect of Sample Preparation on the Detection and Quantification of Selected Nuts Allergenic Proteins by LC-MS/MS
Source: Molecules. 2021 Aug 3;26(15):4698. doi: 10.3390/molecules26154698 (PMC8348345; doi:10.3390/molecules26154698)
Supplement: Supplementary file 1 [file molecules-26-04698-s001.zip › molecules-1313802-supplementary.pdf]

Supplementary Material

Effect of sample preparation on the detection and quantification of selected nuts allergenic proteins by LC-MS/MS

Sorel Tchewonpi Sagu [1], Gerd Huschek [2], Thomas Homann [1] and Harshadrai M. Rawel [1*]

1. University of Potsdam. Institute of Nutritional Science. Arthur-Scheunert-Allee 114-116. 14558 Nuthetal. Potsdam. Germany; sorelsagu@uni-potsdam.de (S.T.S.); thomas.homann@uni-potsdam.de (T.H.)
2. IGV-Institut für Getreideverarbeitung GmbH. Arthur-Scheunert-Allee 40/41. D-14558. Nuthetal OT Bergholz-Rehbrücke. Germany; gerd.huschek@igv-gmbh.de (G.H.)

* Corresponding author.

Tel: +49-33200-88-5525/5578 (H.R.)

E-mail: rawel@uni-potsdam.de

__Content__

Figure S1. Extracted protein (a) and protein extraction yield of nut samples almond, cashew, hazelnut, peanut, pistachio and walnut using Ambi/urea, CM/NaCl, PVPP, SDS and TCA methods

Figure S2, Relative composition of extracted proteins of (a) almond, cashew, hazelnut, and (b) peanut, pistachio and walnut using Ambi/urea, CM/NaCl, PVPP, SDS and TCA methods. The composition of proteins was fragmented in four ranges: 0 – 15 kDa, 15 – 35 kDa, 35 – 70 kDa and 70 – 250 kDa. The volume tools of Image Lab software was used to determine the intensities applying the local subtraction method for the background

Figure S3. Linearity of the HPLC-MS/MS measurements of internal standards (a) GWGG and (b) DPLNVLKPR

Figure S4. Linearity of the HPLC-MS/MS measurements of analyzed almond allergenic proteins (a) Cysteine rich antimicrobial protein (A0A516F3L2), (b) Prunin 1 Pru du 6.0101 (E3SH28), (c) Profilin (Q8GSL5) and (d) (R)-mandelonitrile lyase 2 (Q945K2)

Figure S5. Linearity of the HPLC-MS/MS measurements of analyzed cashew allergenic proteins (a) 11S globulin seed storage protein Ana o 2.0101 Fragment (Q8GZP6), (b) 2s albumin (Q8H2B8) and (c) Vicilin-like protein (Q8L5L5)

Figure S6. Linearity of the HPLC-MS/MS measurements of analyzed hazelnut allergenic proteins (a) Isoflavone reductase (A0A0U1VZC8), (b) 2S albumin (D0PWG2), (c) Vicilin Cor a 11.0101 (Q8S4P9) and (d) Non-specific lipid-transfer protein Cor a 8.0101 (Q9ATH2).

Figure S7. Linearity of the HPLC-MS/MS measurements of analyzed peanut allergenic proteins (a) Non-specific lipid-transfer protein (B6CEX8), (b) Conglutin-7 (Q6PSU2), (c) Conglutin (Q647G9) and (d) Glycinin Fragment (O82580).

Figure S8. Linearity of the HPLC-MS/MS measurements of analyzed pistachio allergenic proteins (a) Superoxide dismutase (B2BDZ8), (b) Pis v 1 allergen 2S albumin (B7P072), (c) Pis v 2.0101 allergen11S globulin precusor (B7P073) and (d) Pis v 5.0101 allergen 11S globulin precusor (B7SLJ1)

Figure S9. Linearity of the HPLC-MS/MS measurements of analyzed walnut allergenic proteins (a) 11S globulin (A0A1L6K371), (b) Vicilin Jug r 6.0101 (A0A2I4E5L6), (c) 2S albumin seed storage protein Fragment (P93198) and (d) 11S globulin seed storage protein Jug r 4 (Q2TPW5)

Figure S10. Recovery of the injected internal standards (a) GWGG and (b) DPLNV(d8)LKPR in the different nut matrixes according to the extraction protocols: ammonium bicarbonate/urea (Ambi/urea), chloroform/methanol/sodium chloride (CM/NaCl), polyvinylpolypyrrolidone (PVPP), sodium dodecyl sulfate (SDS) and trichloroacetic acid (TCA). The results are expressed as ng internal standard detected (n=3)

Figure S11 Sum of the relative amount of selected allergenic proteins of (a) almond, (b) cashew, (c) hazelnut, (d) peanut, (e) pistachio and (f) walnut expressed as peak area per mg of defatted nut flour according to the different extractions protocols: ammonium bicarbonate/urea (Ambi/urea), chloroform/methanol/sodium chloride (CM/NaCl), polyvinylpolypyrrolidone (PVPP), Sodium dodecyl sulfate (SDS) and trichloroacetic acid (TCA). The quantification was performed using the different biomarkers for each protein as determined through method development. Overnight tryptic conventional digestion was performed for all the samples and the experiments were carried out in triplicate (N=3).

Figure S12 Sum of the relative amount of selected allergenic proteins of (a) almond, (b) cashew, (c) hazelnut, (d) peanut, (e) pistachio and (f) walnut expressed as peak area per mg of defatted nut flour according to the different digestion protocols (conventional and microwave assisted tryptic digestion). The quantification was performed using the different biomarkers for each protein as

determined through method development. The best extraction process was used for each nut sample (N=3).

Table S1. List of nut allergenic proteins available from the online database ALLERGEN NOMENCLATURE, the WHO/IUIS Allergen Nomenclature Sub-Committee (http:// www.allergen.org; last accessed 13.06.2021). The information on the sequence of the primary chains and the pI were collected from the online database online database UniProtKB. MW is the molecular weight and AA is the number of amino acid residues.

Table S2. The optimized conditions of the HPLC-MS/MS method for the analysis and relative quantification of almond, cashew, hazelnut, peanut, pistachio and walnut allergenic proteins

Table S3. LC-MS/MS method validation results of almond allergenic proteins

Table S4. LC-MS/MS method validation results of cashew allergenic proteins

Table S5. LC-MS/MS method validation results of hazelnut allergenic proteins

Table S6. LC-MS/MS method validation results of peanut allergenic proteins

Table S7. LC-MS/MS method validation results of pistachio allergenic proteins

Table S8. LC-MS/MS method validation results of walnut allergenic proteins

Table S9. LC-MS/MS method validation results of two peptides used as internal standards

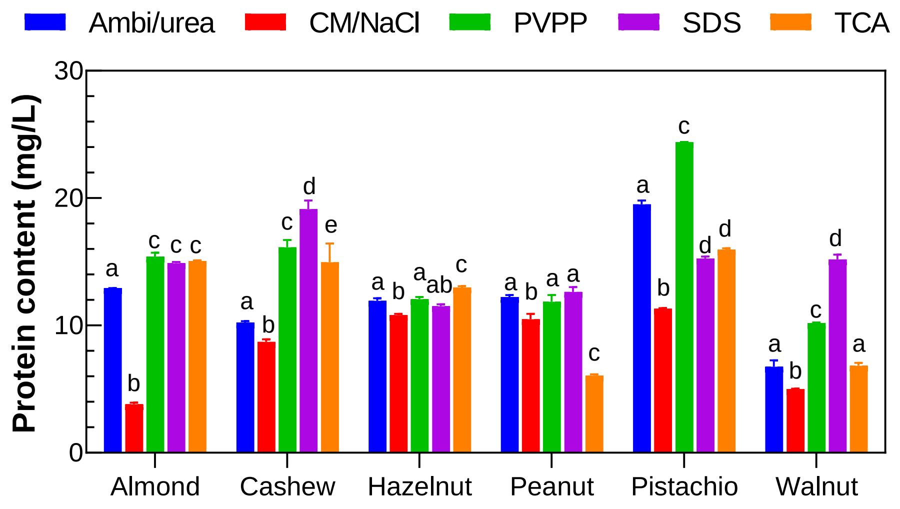

(**a**)

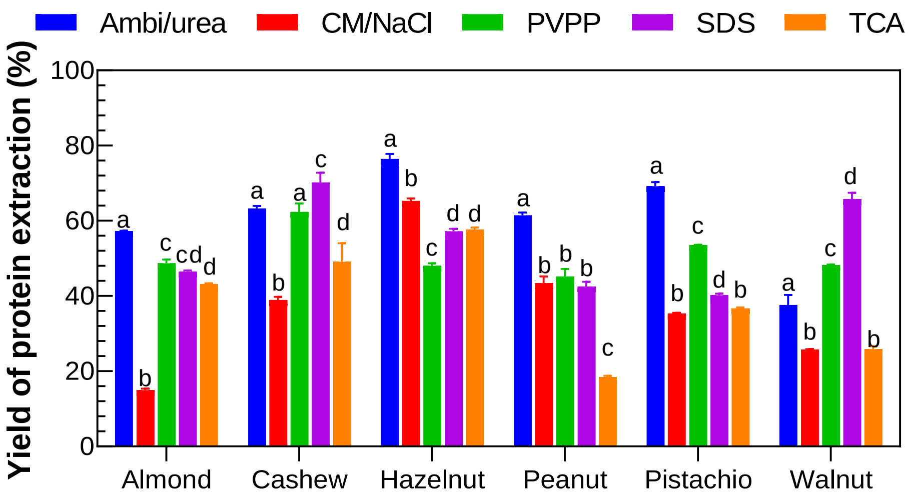

(**b**)

Figure S1. Extracted protein (a) and protein extraction yield of nut samples almond, cashew, hazelnut, peanut, pistachio and walnut using Ambi/urea, CM/NaCl, PVPP, SDS and TCA methods. Different letters indicating statistically significant differences at p=0.05 (Tukey test).

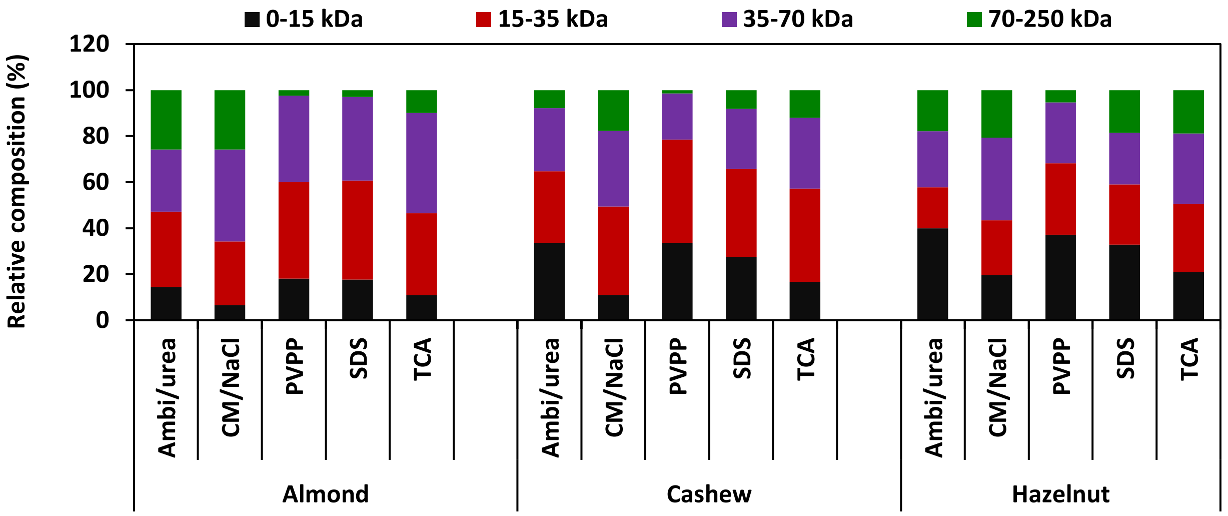

(a)

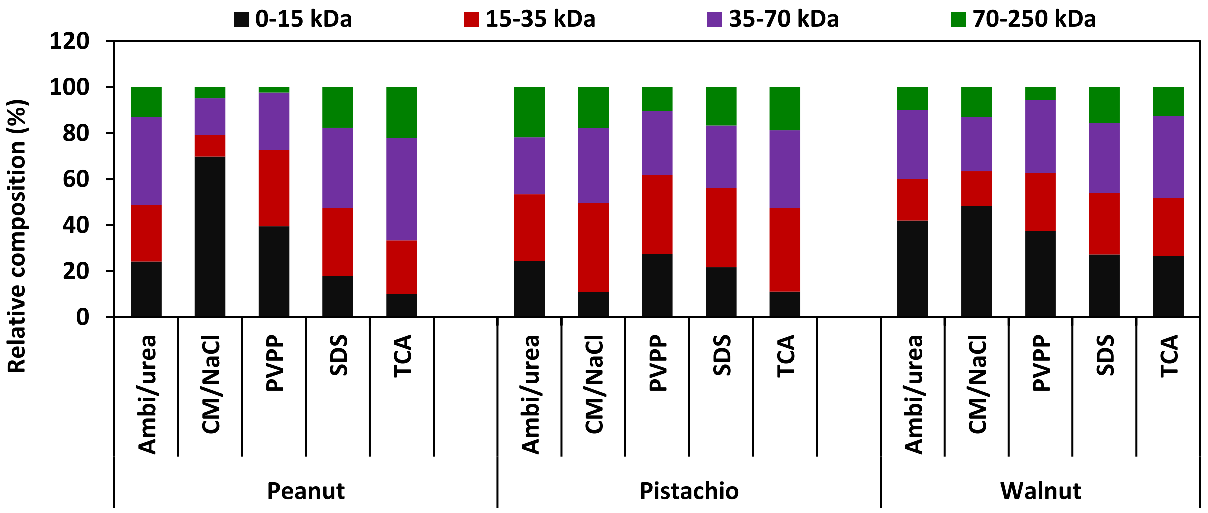

(b)

Figure S2. Relative composition of extracted proteins of (a) almond, cashew, hazelnut, and (b) peanut, pistachio and walnut using Ambi/urea, CM/NaCl, PVPP, SDS and TCA methods. The composition of proteins was fragmented in four ranges: 0 – 15 kDa, 15 – 35 kDa, 35 – 70 kDa and 70 – 250 kDa. The volume tools of Image Lab software was used to determine the intensities applying the local subtraction method for the background.

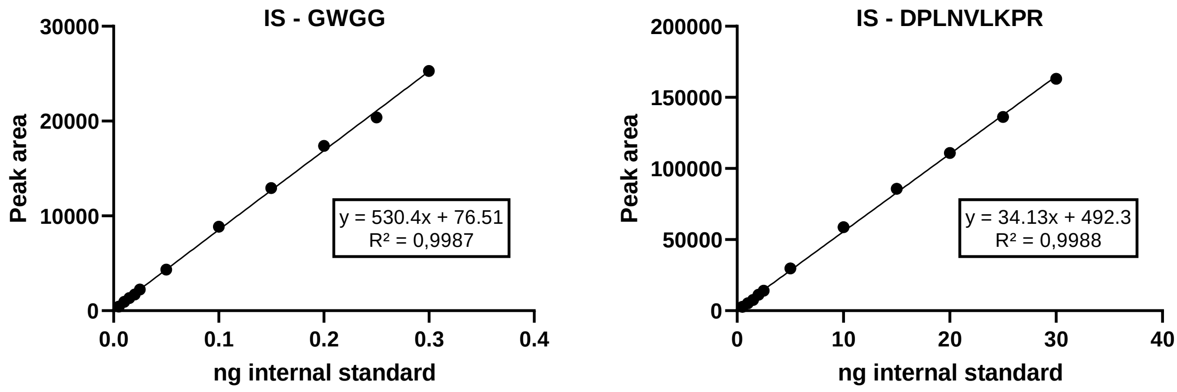

(**a**) (**b**)

Figure S3. Linearity of the HPLC-MS/MS measurements of internal standards (a) GWGG and (b) DPLNVLKPR

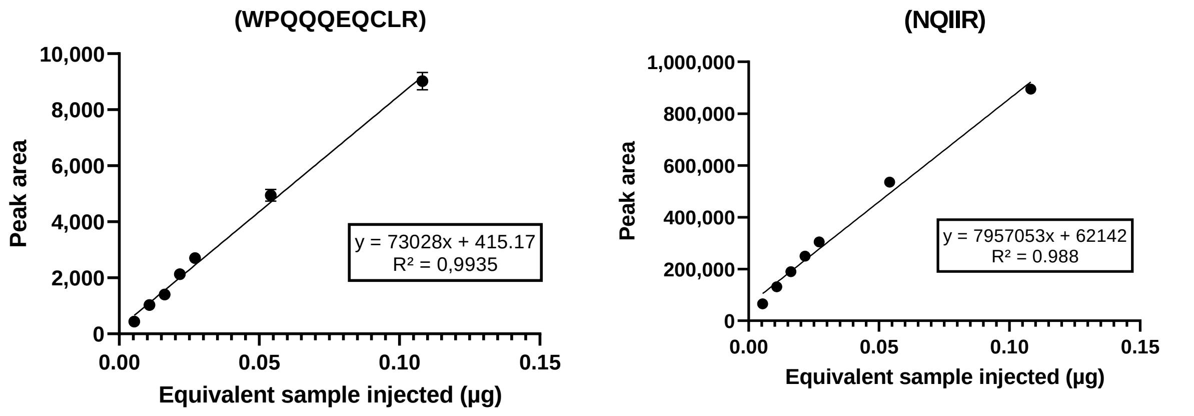

(**a**) (**b**)

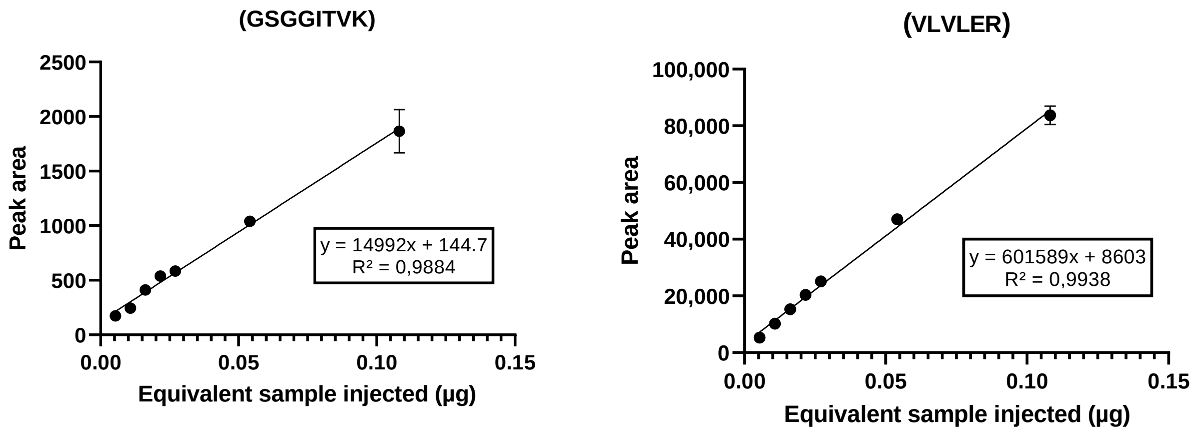

(**c**) (**d**)

Figure S4. Linearity of the HPLC-MS/MS measurements of analyzed almond allergenic proteins (a) Cysteine rich antimicrobial protein (A0A516F3L2), (b) Prunin 1 Pru du 6.0101 (E3SH28), (c) Profilin (Q8GSL5) and (d) (R)-mandelonitrile lyase 2 (Q945K2)

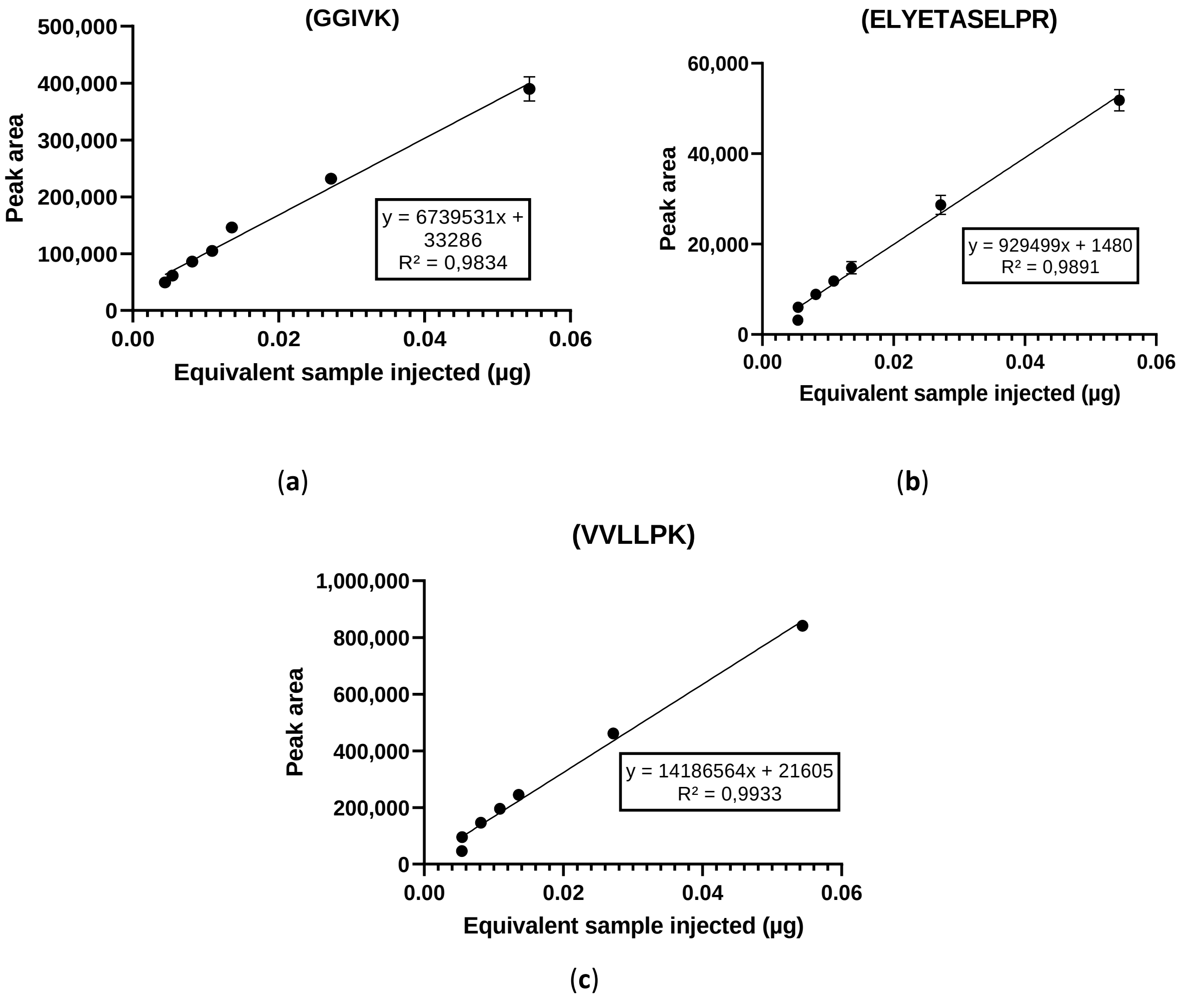

Figure S5. Linearity of the HPLC-MS/MS measurements of analyzed cashew allergenic proteins (a) 11S globulin seed storage protein Ana o 2.0101 Fragment (Q8GZP6), (b) 2s albumin (Q8H2B8) and (c) Vicilin-like protein (Q8L5L5)

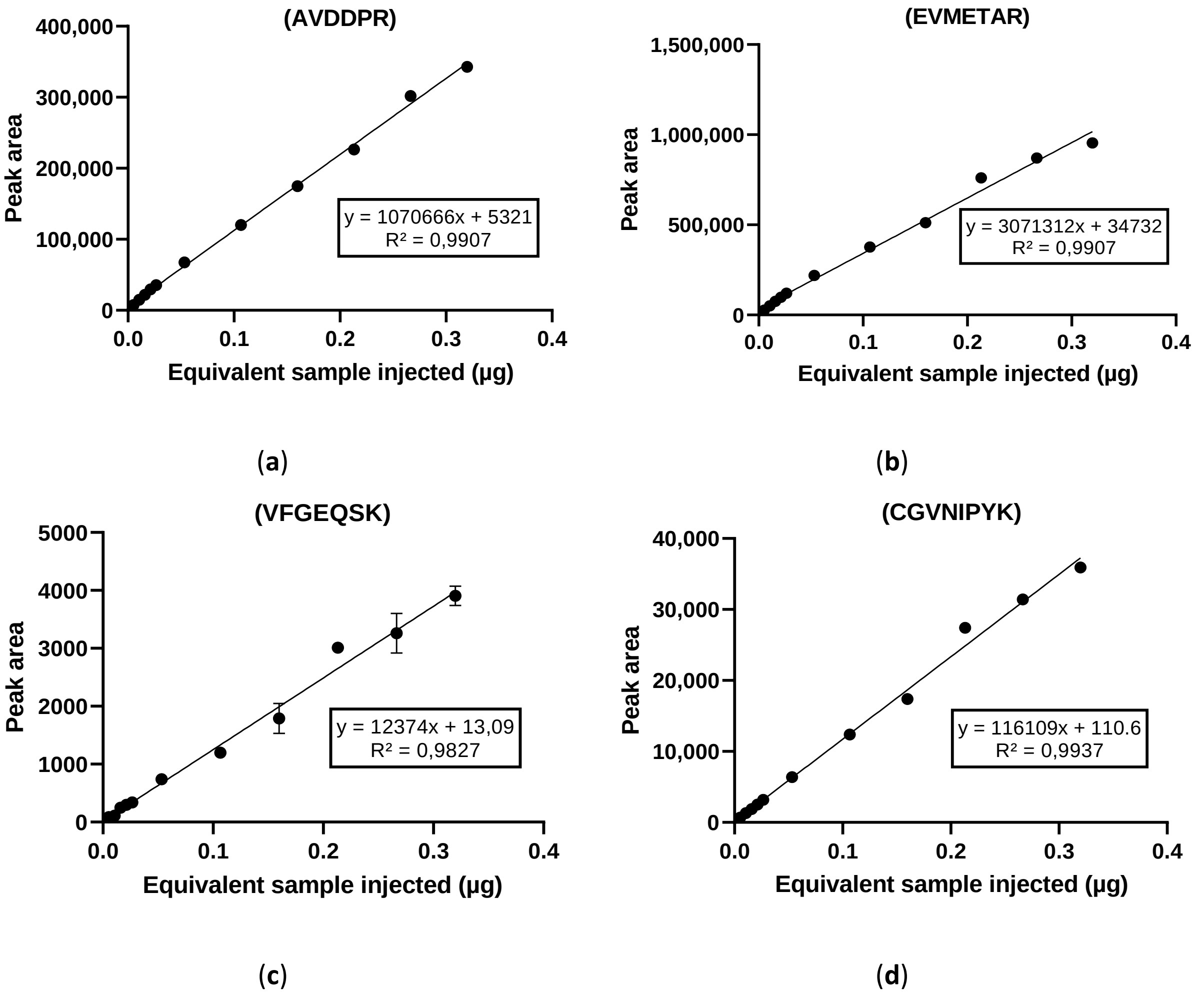

Figure S6. Linearity of the HPLC-MS/MS measurements of analyzed hazelnut allergenic proteins (a) Isoflavone reductase (A0A0U1VZC8), (b) 2S albumin (D0PWG2), (c) Vicilin Cor a 11.0101 (Q8S4P9) and (d) Non-specific lipid-transfer protein Cor a 8.0101 (Q9ATH2).

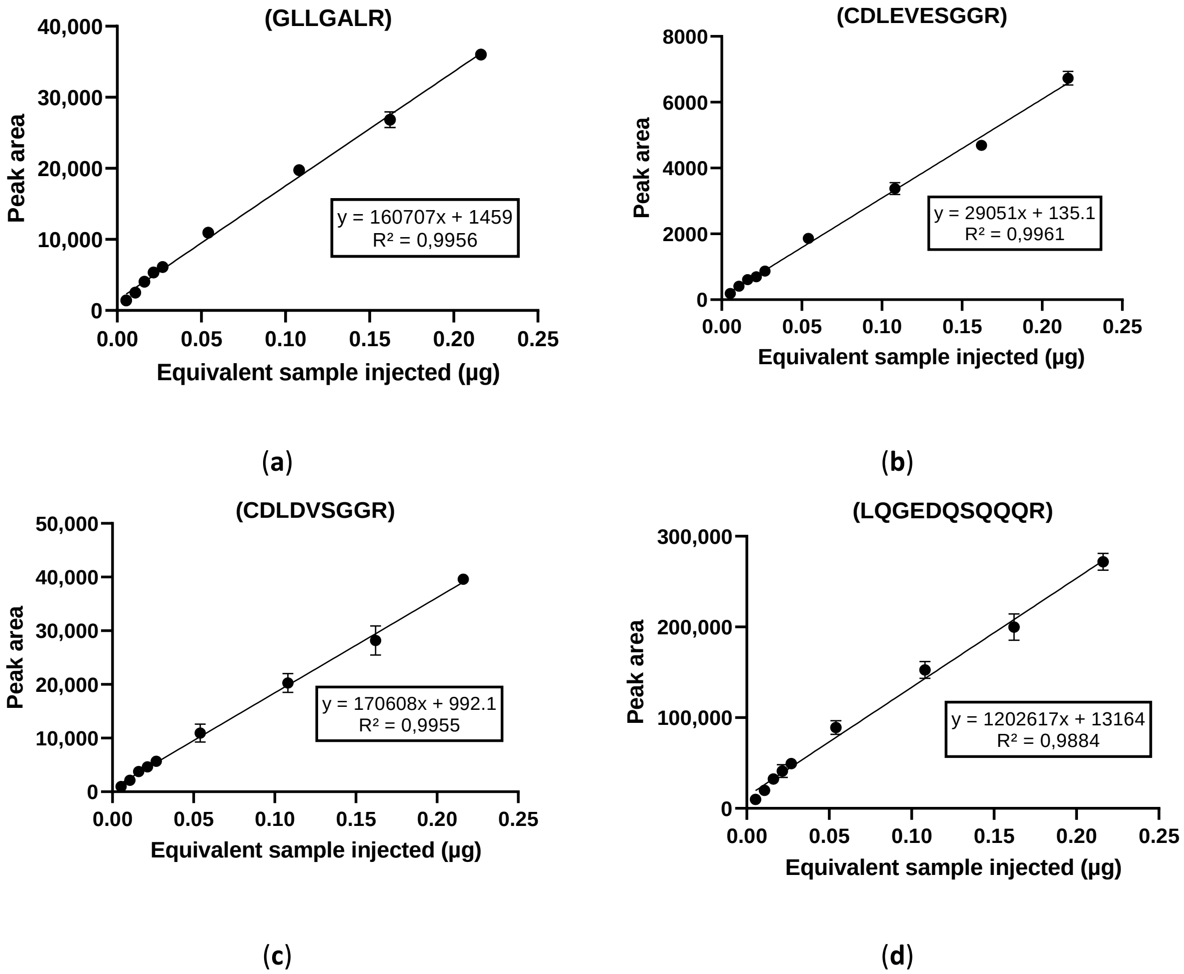

Figure S7. Linearity of the HPLC-MS/MS measurements of analyzed peanut allergenic proteins (a) Non-specific lipid-transfer protein (B6CEX8), (b) Conglutin-7 (Q6PSU2), (c) Conglutin (Q647G9) and (d) Glycinin Fragment (O82580).

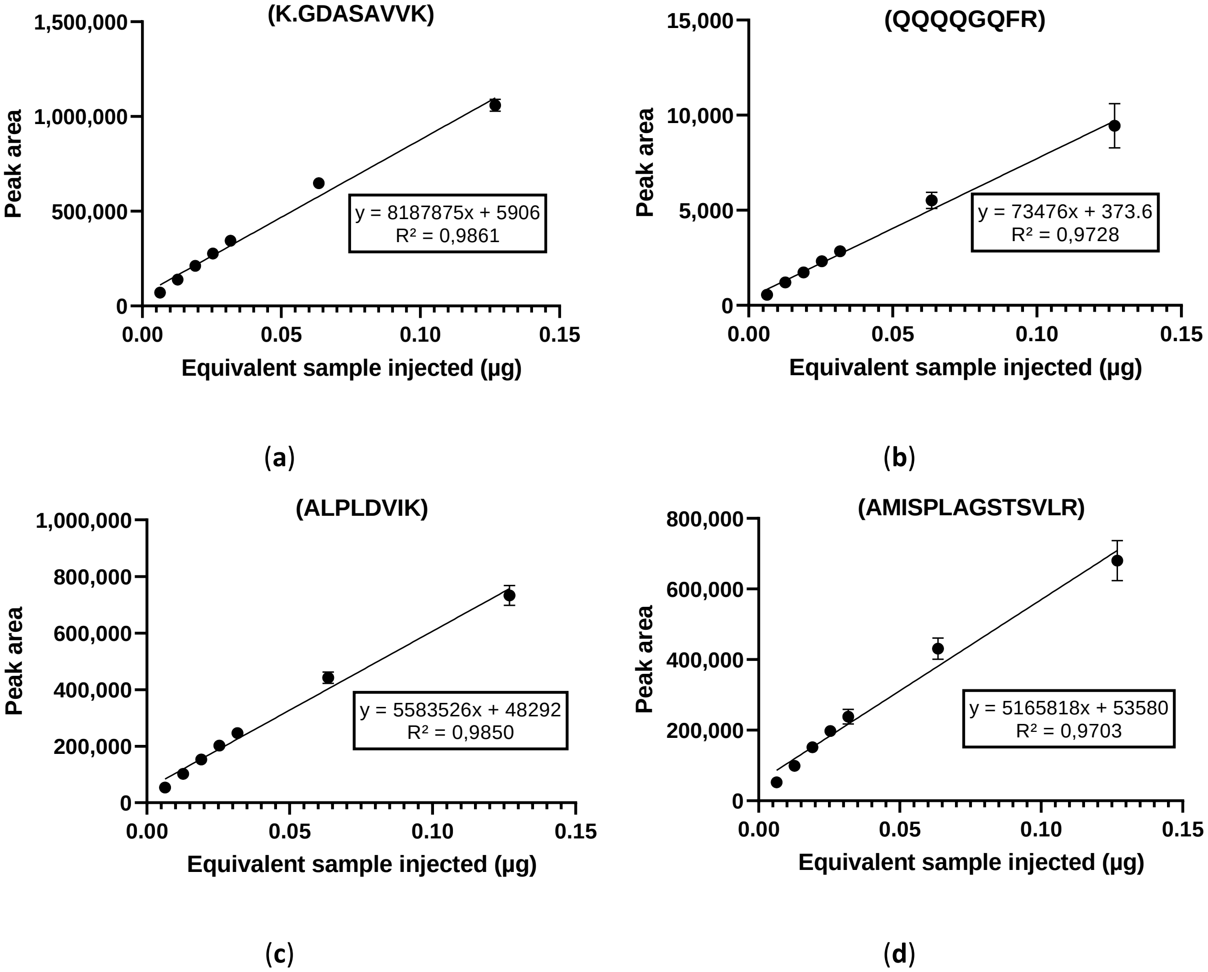

Figure S8. Linearity of the HPLC-MS/MS measurements of analyzed pistachio allergenic proteins (a) Superoxide dismutase (B2BDZ8), (b) Pis v 1 allergen 2S albumin (B7P072), (c) Pis v 2.0101 allergen11S globulin precusor (B7P073) and (d) Pis v 5.0101 allergen 11S globulin precusor (B7SLJ1)

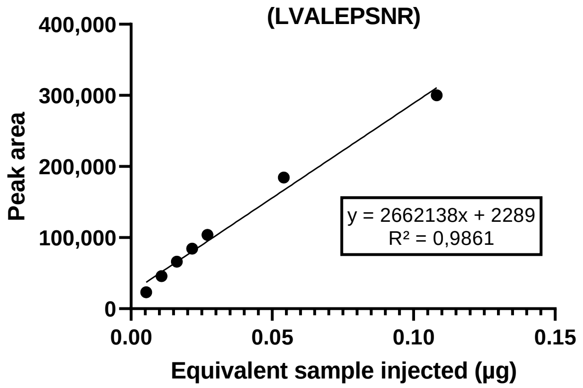

(a)

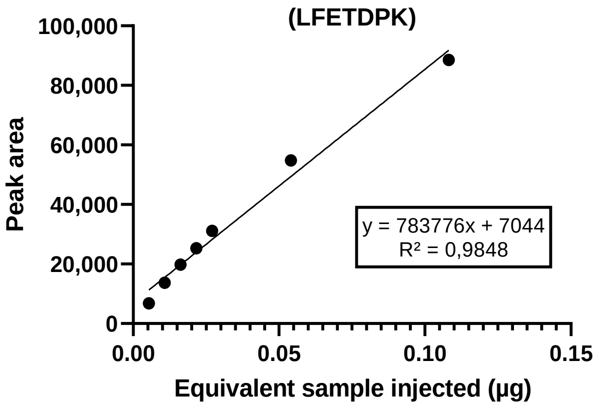

(b)

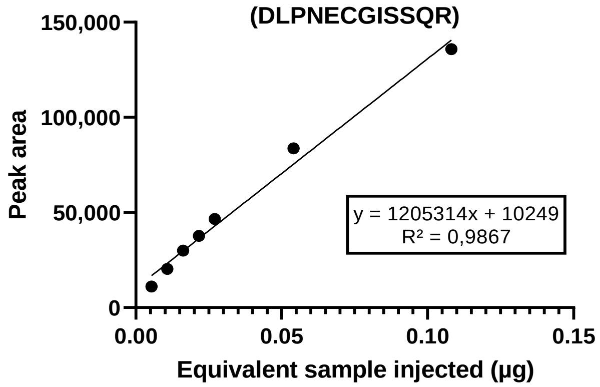

(c)

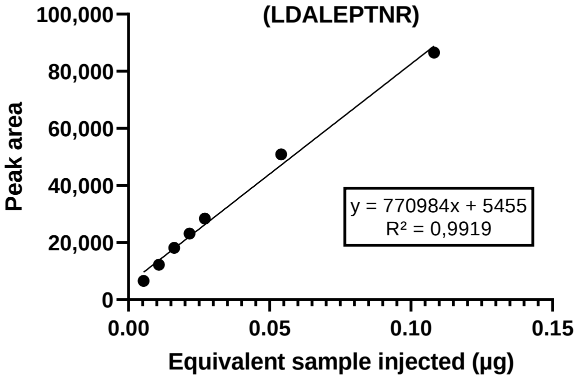

(d)

Figure S9. Linearity of the HPLC-MS/MS measurements of analyzed walnut allergenic proteins (a) 11S globulin (A0A1L6K371), (b) Vicilin Jug r 6.0101 (A0A2I4E5L6), (c) 2S albumin seed storage protein Fragment (P93198) and (d) 11S globulin seed storage protein Jug r 4 (Q2TPW5).

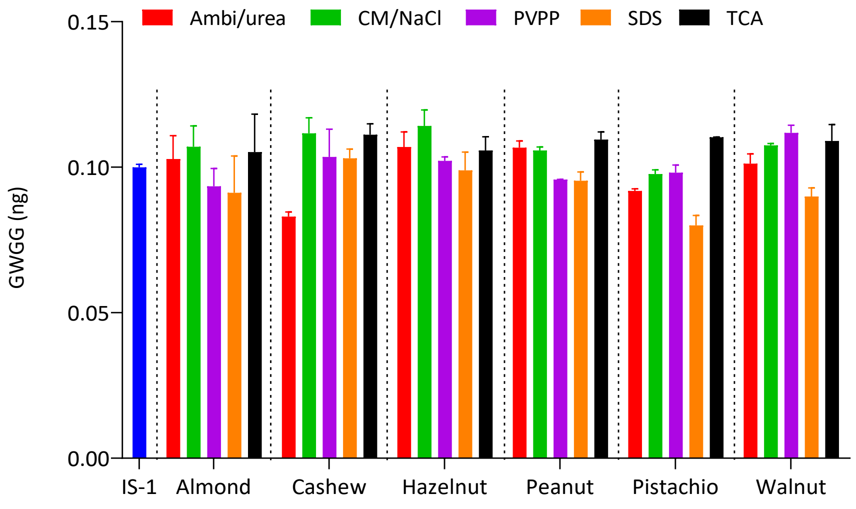

(**a**)

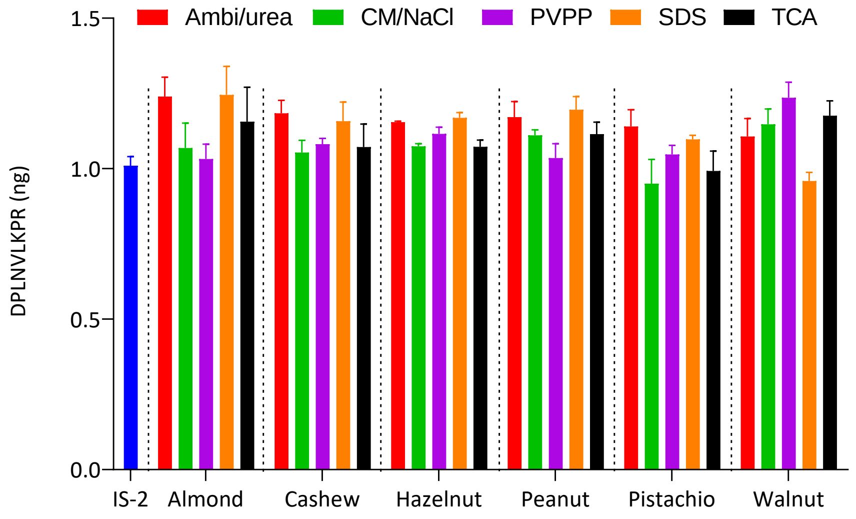

(**b**)

Figure S10. Recovery of the injected internal standards (a) GWGG and (b) DPLNV(d8)LKPR in the different nut matrixes according to the extraction protocols: ammonium bicarbonate/urea (Ambi/urea), chloroform/methanol/sodium chloride (CM/NaCl), polyvinylpolypyrrolidone (PVPP), sodium dodecyl sulfate (SDS) and trichloroacetic acid (TCA). The results are expressed as ng internal standard detected (n=3).

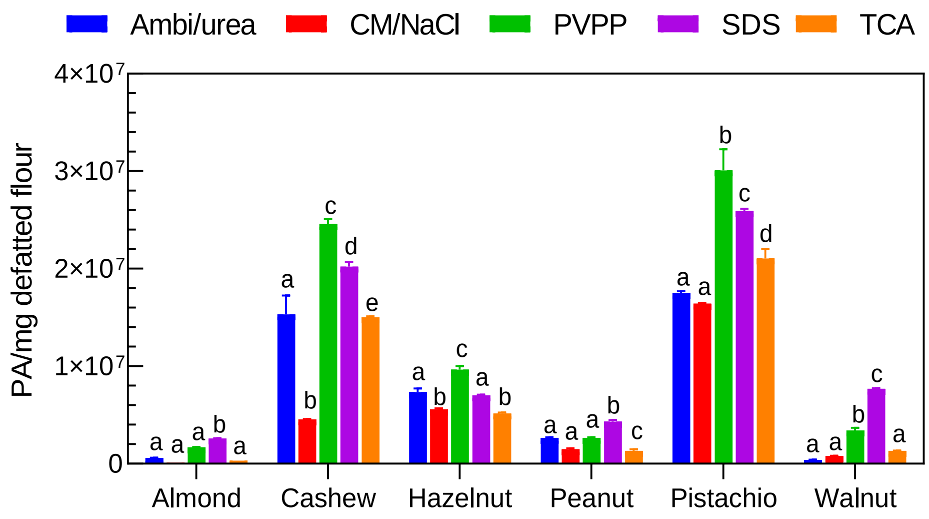

Figure S11 Sum of the relative amount of selected allergenic proteins of (a) almond, (b) cashew, (c) hazelnut, (d) peanut, (e) pistachio and (f) walnut expressed as peak area per mg of defatted nut flour according to the different extractions protocols: ammonium bicarbonate/urea (Ambi/urea), Chloroform/methanol/sodium chloride (CM/NaCl), polyvinylpolypyrrolidone (PVPP), Sodium dodecyl sulfate (SDS) and trichloroacetic acid (TCA). The quantification was performed using the different biomarkers for each protein as determined through method development. Overnight tryptic conventional digestion was performed for all the samples and the experiments were carried out in triplicate (N=3). Different letters indicating statistically significant differences at p=0.05 (Tukey test).

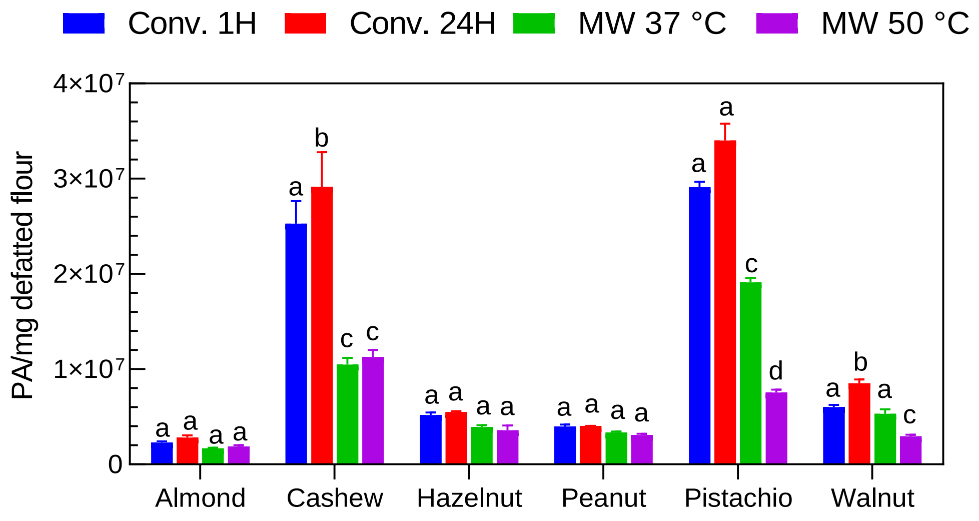

Figure S12 Sum of the relative amount of selected allergenic proteins of (a) almond, (b) cashew, (c) hazelnut, (d) peanut, (e) pistachio and (f) walnut expressed as peak area per mg of defatted nut flour according to the different digestion protocols (conventional and microwave assisted tryptic digestion). The quantification was performed using the different biomarkers for each protein as determined through method development. The best extraction process was used for each nut sample (N=3). Different letters indicating statistically significant differences at p=0.05 (Tukey test).

Table S1. List of nut allergenic proteins available from the online database ALLERGEN NOMENCLATURE, the WHO/IUIS Allergen Nomenclature Sub-Committee (http:// www.allergen.org; last accessed 13.06.2021). The information on the sequence of the primary chains was collected from the online database online database UniProtKB. AA is the number of amino acid residues.

Nut sample	Allergen	Biochemical name	Route of Allergen Exposure	Uniprot entry	Molecular Weight	Length (AA)
**Almond**	Pru du 3	Non-specific lipid transfer protein 1 (nsLTP1)	Food	C0L0I5	9 kDa	102
	Pru du 4	Profilin	Food	Q8GSL5	14 kDa	130
	Pru du 5	60s acidic ribosomal prot. P2	Food	Q8H2B9	10 kDa	113
	Pru du 6	Amandin 11S globulin legumin-like protein	Food	E3SH28	ca. 360 kDa	551
	Pru du 8	Antimicrobial seed storage protein	Food	A0A516F3L2	31 kDa	264
	Pru du 10	Mandelonitrile lyase 2	Food	Q945K2	60 kDa	563
**Cashew**	Ana o 1	Vicilin-like protein	Food	Q8L5L5	50 kDa	538
	Ana o 2	Legumin-like protein	Food	Q8GZP6	55 kDa	457
	Ana o 3	2S albumin	Food	Q8H2B8	14 kDa	138
**Hazelnut**	Cor a 1	Pathogenesis-related protein. PR-10. Bet v 1 family member	Airway	Q08407	17 kDa	160
	Cor a 2	Profilin	Food	Q9AXH5	14 kDa	131
	Cor a 6	Isoflavone reductase homologue	Airway	A0A0U1VZC8	35 kDa	308
	Cor a 8	Non-specific lipid transfer protein type 1	Food	Q9ATH2	9 kDa	115
	Cor a 9	11S seed storage globulin (legumin-like)	Food	Q8W1C2	40 kDa	515
	Cor a 10	Luminal binding protein	Airway	Q9FSY7	70 kDa	668
	Cor a 11	7S seed storage globulin (vicilin-like)	Food	Q8S4P9	48 kDa	448
	Cor a 12	17 kDa oleosin	Food	Q84T21	17 kDa	159
	Cor a 13	14-16 kDa oleosin	Food	Q84T91	14-16 kDa	140
	Cor a 14	2S albumin	Food	D0PWG2	10 kDa reducing	147

Table S1. Continued

Nut sample	Allergen	Biochemical name	Route of Allergen Exposure	Uniprot entry	MW (Da)	Length (AA)
Peanut	Ara h 1	Cupin (Vicillin-type. 7S globulin)		P43238	64 kDa	626
	Ara h 2	Conglutin (2S albumin)	Food	Q6PSU2	17 kDa	172
	Ara h 3	Cupin (Legumin-type. 11S globulin. Glycinin)	Food	O82580	60 kDa. 37 kDa (fragment)	507
	Ara h 4	renamed to Ara h 3.02. number not available for future submissions	Food	Q9SQH7	61.1 kDa	530
	Ara h 5	Profilin	Food	Q9SQI9	15 kDa	131
	Ara h 6	Conglutin (2S albumin)	Food	Q647G9	15 kDa	145
	Ara h 7	Conglutin (2S albumin)	Food	Q9SQH1	15 kDa	160
	Ara h 8	Pathogenesis-related protein. PR-10. Bet v 1 family member	Food	Q6VT83	17 kDa	157
	Ara h 9	Nonspecific lipid-transfer protein type 1	Food	B6CEX8	9.8 kDa	116
	Ara h 10	oleosin	Food	Q647G5	16 kDa	169
	Ara h 11	oleosin	Food	Q45W87	14 kDa	137
	Ara h 12	Defensin	Food	B3EWP3	8 kDa (reducing). 12 kDa (non-reducing). 5.184 kDa (mass)	71
	Ara h 13	Defensin	Food	B3EWP4	8 kDa (reducing). 11 kDa (non-reducing). 5.472 kDa (mass)	79
	Ara h 14	Oleosin	Food	Q9AXI1	17.5 kDa	176
	Ara h 15	Oleosin	Food	Q647G3	17 kDa	166
	Ara h 16	non-specific Lipid Transfer Protein 2	Food	A0A509ZX51	8.5 kDa by SDS PAGE reducing	68
	Ara h 17	non-specific Lipid Transfer Protein 1	Food	A0A510A9S3	11 kDa by SDS-PAGE reducing	93
	Ara h 18	Cyclophilin - peptidyl-prolyl cis-trans isomerase	Food	A0A444XS96	21 kDa	226

Table S1. Continued

Nut sample	Allergen	Biochemical name	Route of Allergen Exposure	Uniprot entry	MW (Da)	Length (AA)
**Pistachio**	Pis v 1	2S albumin	Food	B7P072	7 kDa	149
	Pis v 2	11S globulin subunit	Food	B7P073	32 kDa	496
	Pis v 3	vicillin	Food	B4X640	55 kDa	545
	Pis v 4	manganese superoxide dismutase	Food	B2BDZ8	25.7 kDa	230
	Pis v 5	11S globulin subunit	Food	B7SLJ1	36 kDa (acidic subunit)	473
**Black Walnut**	Jug n 1	2S albumin seed storage protein	Food	Q7Y1C2	18.9 kDa	161
	Jug n 2	Vicilin seed storage protein	Food	Q7Y1C1	55.6 kDa	481
	Jug n 4	Legumin	Food	A0A1L6K371	58.1 kDa	510
**English walnut**	Jug r 1	2S albumin seed storage protein	Food	P93198	15-16 kDa	
	Jug r 2	Vicilin seed storage protein	Food	Q9SEW4	44 kDa	139
	Jug r 3	Non-specific lipid transfer protein type 1 (nsLTP1)	Food	C5H617	9 kDa	119
	Jug r 4	11S globulin seed storage protein	Food	Q2TPW5	58.1 kDa	507
	Jug r 5	PR-10	Food	-	20 kDa	-
	Jug r 6	vicilin-like cupin	Food	A0A2I4E5L6	47 kDa	502
	Jug r 7	Profilin	Food	A0A2I4DNN6	13 kDa	131
	Jug r 8	ns-LTP-2	Food	-	9 kDa	-

Table S2. The optimized conditions of the HPLC-MS/MS method for the analysis and relative quantification of almond, cashew, hazelnut, peanut, pistachio and walnut allergenic proteins.

	Allergenic protein	Biomarker peptide	Precursor Ion	Fragment	Product Ion	Collision Energy	Retention time (min)
Almond	A0A516F3L2	WPQQQEQC[+57.021464]LR.light	686.82496	Q [y6]	833.393412	28.3	9.6
				E [y5]	705.334835	31.3	
				Q [y4]	576.292242	31.3	
				P [b2]	284.139353	31.3	
	E3SH28	NQIIR.light	322.19793	I [y3]	401.28708	8	7.8
				I [y2]	288.203016	17	
				Q [b2]	243.108781	8	
				R [y1]	175.118952	20	
	Q8GSL5	GSGGITVK.light	359.708328	G [y5]	517.334424	15.2	9.9
				T [y3]	347.228896	21.2	
				G [b4]	259.103696	12.2	
				V [y2]	246.181218	21.2	
	Q945K2	VLVLER.light	364.736888	L [y5]	629.398087	15.3	10.2
				V [y4]	516.314023	12.3	
				L [y3]	417.245609	9.3	

Table S2. Continued

	Allergenic protein	Biomarker peptide	Precursor Ion	Fragment	Product Ion	Collision Energy	Retention time (min)
Cashew	Q8GZP6	GGIVK.light	237.157743	I [y3]	359.265282	5.4	6.8
				V [y2]	246.181218	8.4	
				K [y1]	228.134267	5.4	
				I [b3]	147.112804	11.4	
	Q8H2B8	ELYETASELPR.light	654.327524	T [y7]	773.415194	21.3	10.5
				A [y6]	672.367515	21.3	
				S [y5]	601.330401	18.3	
				P [y2]	272.171716	15.3	
	Q8H2B8	AIVEVEEDSGR.light	602.296224	E [y6]	692.284573	16.7	12.4
				S [y3]	413.239461	19.7	
				G [y2]	319.172444	16.7	
				E [b4]	232.140415	25.7	
	Q8L5L5	VVLLPK.light	334.7389	V [y5]	569.40211	11.4	10.4
				L [y4]	470.333696	8.4	
				P [y2	244.165568	14.4	
Hazelnut	A0A0U1VZC8	AVDDPR.light	336.669203	P [y2]	498.219454	20.4	9.9
				R [y1]	272.171716	20.4	
				P [b5]	175.118952	20.4	
	D0PWG2	EVMETAR.light	418.202552	M [y5]	607.286822	14	7.4
				E [y4]	476.246337	14	
				T [y3]	347.203744	11	
				A [y2]	246.156066	11	
	Q8S4P9	VFGEQSK.light	397.705785	F [y6]	695.335881	13.3	7.8
				G [y5]	548.267467	13.3	
				S [y2]	234.144832	22.3	

Table S2. Continued

	Allergenic protein	Biomarker peptide	Precursor Ion	Fragment	Product Ion	Collision Energy	Retention time (min)
**Hazelnut**	Q9ATH2	C[+57.021464]GVNIPYK.light	475.741845	N [y5]	634.355888	18.7	9.8
				P [y3]	407.228896	18.7	
				G [b2]	317.127802	24.7	
				V [b3]	218.059388	18.7	
**Peanut**	B6CEX8	GLLGALR.light	350.229231	R [y1]	341.218332	5.9	10.6
				L [b3]	284.196868	11.9	
				G [b4]	175.118952	20.9	
	O82580	LQGEDQSQQQR.light	658.813104	Q [y4]	559.294684	27.4	10.9
				G [b3]	543.240917	24.4	
				D [b5]	299.171381	15.4	
	Q647G9	CDLDVSGGR.light	489.7191	L [y7]	703.3733	16.2	8.4
				D [y6]	590.2893	19.2	
				V [y5]	475.2623	19.2	
				S [y4]	376.1939	16.2	
	Q6PSU2	C[+57.021464]DLEVESGGR.light	561.24822	E [y7]	733.347508	24.4	8.6
				V [y6]	604.304915	24.4	
				E [y5]	505.236501	21.4	
				L [b3]	389.148932	24.4	
**Pistachio**	B2BDZ8	GDASAVVK.light	373.705785	A [y6]	574.355888	12.6	7.8
				S [y5]	503.318774	12.6	
				A [y4]	416.286745	12.6	
				V [y2]	246.181218	12.6	
	B7P072	QQQQGQFR.light	510.254497	Q [y5]	635.325985	16.8	7.0
				G [y4]	507.267407	16.8	
				F [y2]	322.187366	16.8	

Table S2. Continued

	Allergenic protein	Biomarker peptide	Precursor Ion	Fragment	Product Ion	Collision Energy	Retention time (min)
Pistachio	B7P073	ALPLDVIK.light	434.778753	P [y6]	684.429053	14.5	12.1
				L [y5]	587.376289	14.5	
				D [y4]	474.292225	14.5	
				I [y2]	260.196868	14.5	
	B7SLJ1	AMISPLAGSTSVLR.light	701.889769	P [y10]	1000.578571	22.8	12.3
				M [b2]	403.200967	22.8	
				I [b3]	316.168938	22.8	
				S [b4]	203.084874	22.8	
Walnut	A0A1L6K371	LVALEPSNR.light	499.785098	A [y7]	786.410442	19.5	9.7
				E [y5]	602.289265	13.5	
				P [y4]	473.246672	22.5	
				A [b3]	284.196868	16.5	
	A0A2I4E5L6	LFETDPK.light	425.221268	E [y5]	589.282782	11.2	9.2
				P [y2]	244.165568	14.2	
				K [y1]	147.112804	23.2	
	P93198	DLPNEC[+57.021464]GISSQR.light	688.317165	N [y9]	1050.463283	31.3	9.1
				G [y6]	647.347114	31.3	
				S [y4]	477.241586	31.3	
				R [y1]	175.118952	31.3	
	Q2TPW5	LDALEPTNR.light	514.772188	L [y6]	729.388979	17	9.3
				E [y5]	616.304915	17	
				P [y4]	487.262322	20	
				A [b3]	300.155397	20	

Table S2. Continued

	Allergenic protein	Biomarker peptide	Precursor Ion	Fragment	Product Ion	Collision Energy	Retention time (min)
**Internal standards**	Int-STD	GWGG.light	376.161545	W [y3]	319.140081	9.7	8.3
				G [y2]	301.129516	9.7	
				G [y1]	244.108053	9.7	
				W [b2	133.060768	9.7	
				G [b3]	76.039304	15.7	
	IS FGF23	DPLNVLKPR.heavy	530.347866	P [y8]	944.661512	26.3	10.5
				K [b7]	788.524015	14.3	
				V [y5]	620.481757	23.3	
				V [b5]	547.344988	20.3	
				L [y4]	513.350743	23.3	
				N [b4]	440.213974	23.3	

Table S3. LC-MS/MS method validation results of almond allergenic proteins

Validation criterion	A0A516F3L2 (WPQQQEQCLR)	E3SH28 (NQIIR)	Q8GSL5 (GSGGITVK)	Q945K2 (VLVLER)
Repeatability (% RSD)	3.42	1.19	4.69	3.92
Reproducibility (% RSD)	2.68	1.94	2.38	3.74
Linearity (R^2)	0.9935	0.9880	0.9799	0.9938
LoD (ng flour)	37.947	37.531	38.372	35.604
LoQ (ng flour)	126.489	125.103	127.906	118.681

RSD = relative standard deviation. LoD = limit of detection. LoQ = limit of quantification.

Table S4. LC-MS/MS method validation results of cashew allergenic proteins

Validation criterion	Q8GZP6 (GGIVK)	Q8H2B8 (ELYETASELPR)	Q8L5L5 (VVLLPK)
Repeatability (% RSD)	5.47	4.55	1.92
Reproducibility (% RSD)	6.66	1.20	0.13
Linearity (R^2)	0.9834	0.9875	0.9904
LoD (ng flour)	69.932	29.615	48.371
LoQ (ng flour)	233.107	98.716	161.237

Table S5. LC-MS/MS method validation results of hazelnut allergenic proteins

Validation criterion	A0A0U1VZC8 (AVDDPR)	D0PWG2 (EVMETAR)	Q8S4P9 (VFGEQSK)	Q9ATH2 (CGVNIPYK)
Repeatability (% RSD)	1.25	0.54	0.65	4.49
Reproducibility (% RSD)	1.86	2.39	0.68	1.60
Linearity (R^2)	0.9981	0.9907	0.9824	0.9932
LoD (ng flour)	39.479	18.389	12.305	13.018
LoQ (ng flour)	131.598	61.296	41.018	43.395

Table S6. LC-MS/MS method validation results for the analysis of peanut allergenic proteins

Validation criterion	B6CEX8 (GLLGALR)	Q6PSU2 (CDLEVESGGR)	Q647G9 (CDLDVSGGR)	O82580 (LQGEDQSQQQR)
Repeatability (% RSD)	1.90	5.45	0.23	3.44
Reproducibility (% RSD)	2.02	3.07	6.81	6.10
Linearity (R^2)	0.9956	0.9961	0.9955	0.9884
LoD (ng flour)	29.051	10.745	9.193	19.118
LoQ (ng flour)	96.835	35.815	30.644	63.728

Table S7. LC-MS/MS method validation results of pistachio allergenic proteins

Validation criterion	B4X640 (VVVLPK)	B7P072 (QQQQGQFR)	B7P073 (ALPLDVIK)	B7SLJ1 (AMISPLAGSTSVLR)
Repeatability (% RSD)	2.11	7.85	4.65	6.99
Reproducibility (% RSD)	3.43	6.94	3.23	5.77
Linearity (R^2)	0.9867	0.9728	0.9850	0.9703
LoD (ng flour)	42.757	23.343	35.079	24.031
LoQ (ng flour)	142.522	77.811	116.931	80.104

Table S8. LC-MS/MS method validation results of walnut allergenic proteins

Validation criterion	A0A1L6K371 (LVALEPSNR)	A0A2I4E5L6 (LFETDPK)	P93198 (DLPNECGISSQR)	Q2TPW5 (LDALEPTNR)
Repeatability (% RSD)	1.47	0.13	0.97	1.25
Reproducibility (% RSD)	2.51	2.31	2.63	0.77
Linearity (R^2)	0.9861	0.9848	0.9867	0.9919
LoD (ng flour)	36.549	37.665	34.469	35.881
LoQ (ng flour)	121.831	125.550	114.895	119.605

Table S9. LC-MS/MS method validation results of two peptides used as internal standards

Validation criterion	IS - GWGG	IS - DPLNVLKPR
Repeatability (% RSD)	1.87	0.91
Reproducibility (% RSD)	3.39	4.80
Linearity (R^2)	0.9987	0.9988
LoD (pg peptide)	13.170	11.797
LoQ (pg peptide)	43.900	39.322
